# Association between the total bilirubin to prothrombin time ratio index and diabetic retinopathy, nephropathy, peripheral neuropathy, and foot disease: a retrospective study and risk prediction model construction

**DOI:** 10.3389/fendo.2025.1682680

**Published:** 2026-01-12

**Authors:** Tao Sun, Shuxian Li, Jun Liu

**Affiliations:** 1 Department of Hematology and Oncology Laboratory, The Central Hospital of Shaoyang, Shaoyang, Hunan, China; 2 Department of Intensive Care Unit, The Traditional Chinese Medicine Hospital of Longquanyi, Chengdu, China; 3 Department of Scientific Research, The First Affiliated Hospital of Shaoyang University, Shaoyang, Hunan, China

**Keywords:** diabetic complications, nomogram, prediction model, prothrombin time, total bilirubin, type 2 diabetes mellitus

## Abstract

**Background:**

To investigate the association between the total bilirubin-to-prothrombin time ratio index (TBPTRI) and major chronic complications of type 2 diabetes mellitus (T2DM), including diabetic nephropathy (DN), diabetic retinopathy (DR), diabetic peripheral neuropathy (DPN), and diabetic foot (DF), and to evaluate its predictive value for multiple complications.

**Methods:**

This retrospective cross-sectional study analyzed 15,695 hospitalized T2DM patients at the Central Hospital of Shaoyang from January 2019 to December 2024, with 5,117 eligible patients ultimately included. Missing data were handled using multiple imputation by chained equations (MICE). Logistic regression was employed to assess the independent correlation between TBPTRI and various complications, while restricted cubic spline (RCS) analysis was used to evaluate nonlinear relationships. To enhance model stability and robustness, subgroup and sensitivity analyses were conducted. Feature variables were further screened using the Boruta algorithm, LASSO regression, and Random Forest (RF) to construct a TBPTRI-based complication risk prediction model. A nomogram was developed, and model performance was evaluated using receiver operating characteristic (ROC) curve analysis.

**Results:**

Multivariate logistic regression revealed that TBPTRI was significantly inversely associated with the risks of diabetic nephropathy (OR = 0.61, 95% CI: 0.48–0.79), retinopathy (OR = 0.53, 95% CI: 0.37–0.76), peripheral neuropathy (OR = 0.82, 95% CI: 0.70–0.95), and foot disease (OR = 0.52, 95% CI: 0.40–0.68) (all P < 0.01). RCS analysis indicated nonlinear relationships between TBPTRI and complication risks in diabetic nephropathy and foot disease (P-nonlinear < 0.05). Subgroup analysis demonstrated that the protective effect of TBPTRI remained relatively stable across populations with different clinical characteristics, with significant interactions observed in patients with hypertension or coronary heart disease (CHD). Furthermore, the nomogram constructed using core variables selected by LASSO regression and RF exhibited strong predictive performance, with area under the curve (AUC) values in the test set of 0.871 for nephropathy, 0.647 for retinopathy, 0.735 for peripheral neuropathy, and 0.855 for foot disease.

**Conclusion:**

TBPTRI was inversely associated with major chronic complications of T2DM and demonstrated high predictive value for patients with multiple complications. The TBPTRI-based model exhibited robust performance, supporting its utility in early detection, risk stratification, and precision prevention.

## 1 Introduction

Type 2 diabetes mellitus (T2DM) is a chronic hyperglycemic state characterized by insulin resistance and progressive decline in pancreatic β-cell function (1, 2). Its prevalence has continued to rise in recent years, becoming a major global public health concern (3). According to data released by the International Diabetes Federation (IDF) in 2021, the number of individuals with diabetes worldwide has exceeded 537 million and is projected to reach 783 million by 2045 (4, 5). Notably, T2DM accounts for over 90% of all diabetes cases (6). In China, the prevalence of diabetes has reached 12.4%, with more than 140 million affected individuals, ranking first globally (7). Moreover, the increasing trend of early-onset T2DM poses a significant threat to public health and imposes a substantial burden on the national healthcare system (8, 9).

The detrimental effects of T2DM primarily stem from prolonged hyperglycemia-induced damage to multiple organ systems, particularly the development and progression of chronic complications, including diabetic nephropathy (DN), diabetic retinopathy (DR), diabetic peripheral neuropathy (DPN), and diabetic foot (DF) (10, 11). These complications represent the leading causes of diabetes-related disability and mortality, while also contributing to diminished quality of life and increased healthcare resource utilization (12–14). Therefore, establishing simple, sensitive, and cost-effective risk prediction indicators holds profound clinical and societal significance for the early identification and precise intervention of chronic complications in T2DM.

Oxidative stress and chronic inflammation are recognized as key pathological processes in the pathophysiology of diabetic chronic complications (15, 16). Total bilirubin (TBIL), the end product of heme metabolism, exhibits potent antioxidant and anti-inflammatory properties (17). Extensive epidemiological studies have demonstrated an inverse correlation between TBIL levels and various metabolic disorders, such as atherosclerosis, cardiovascular disease, and chronic kidney disease, suggesting a potential protective role in the pathogenesis of diabetic complications (18–20). However, the sensitivity and specificity of TBIL as a standalone predictive biomarker remain limited. On the other hand, prothrombin time (PT), a representative marker of the extrinsic coagulation pathway, reflects coagulation status, liver function, and systemic inflammation, all of which are closely associated with the hypercoagulable state and microvascular complications in diabetes (21, 22). Integrating TBIL with PT to form TBPTRI may provide a more comprehensive assessment of antioxidant-anti-inflammatory and coagulation-inflammatory states, thereby helping to untangle the underlying pathophysiological mechanisms of diabetic complications.

However, systematic investigations into the association between TBPTRI and major chronic complications of T2DM remains scarce. Existing literature predominantly focuses on the relationship between diabetes complications and conventional metabolic markers, lacking a comprehensive evaluation of integrated biomarkers under multifactorial pathological conditions (23, 24). Moreover, model-based approaches or visualization techniques have been rarely employed to facilitate risk stratification and clinical translation research of TBPTRI. This study retrospectively analyzed real-world clinical data from hospitalized patients at the Central Hospital of Shaoyang between January 2019 and December 2024 to investigate the association between TBPTRI and four major chronic complications of T2DM (DN, DR, DPN, and DF). Restricted cubic spline (RCS) analysis was employed to explore potential nonlinear relationships. Subsequently, LASSO regression, the Boruta algorithm, and Random Forest (RF) were utilized to identify key predictive factors. Risk prediction models centered on TBPTRI were constructed for the four major chronic complications of T2DM (DN, DR, DPN, and DF), with corresponding nomograms developed.

## 2 Materials and methods

### 2.1 Study population

**Figure 1:**
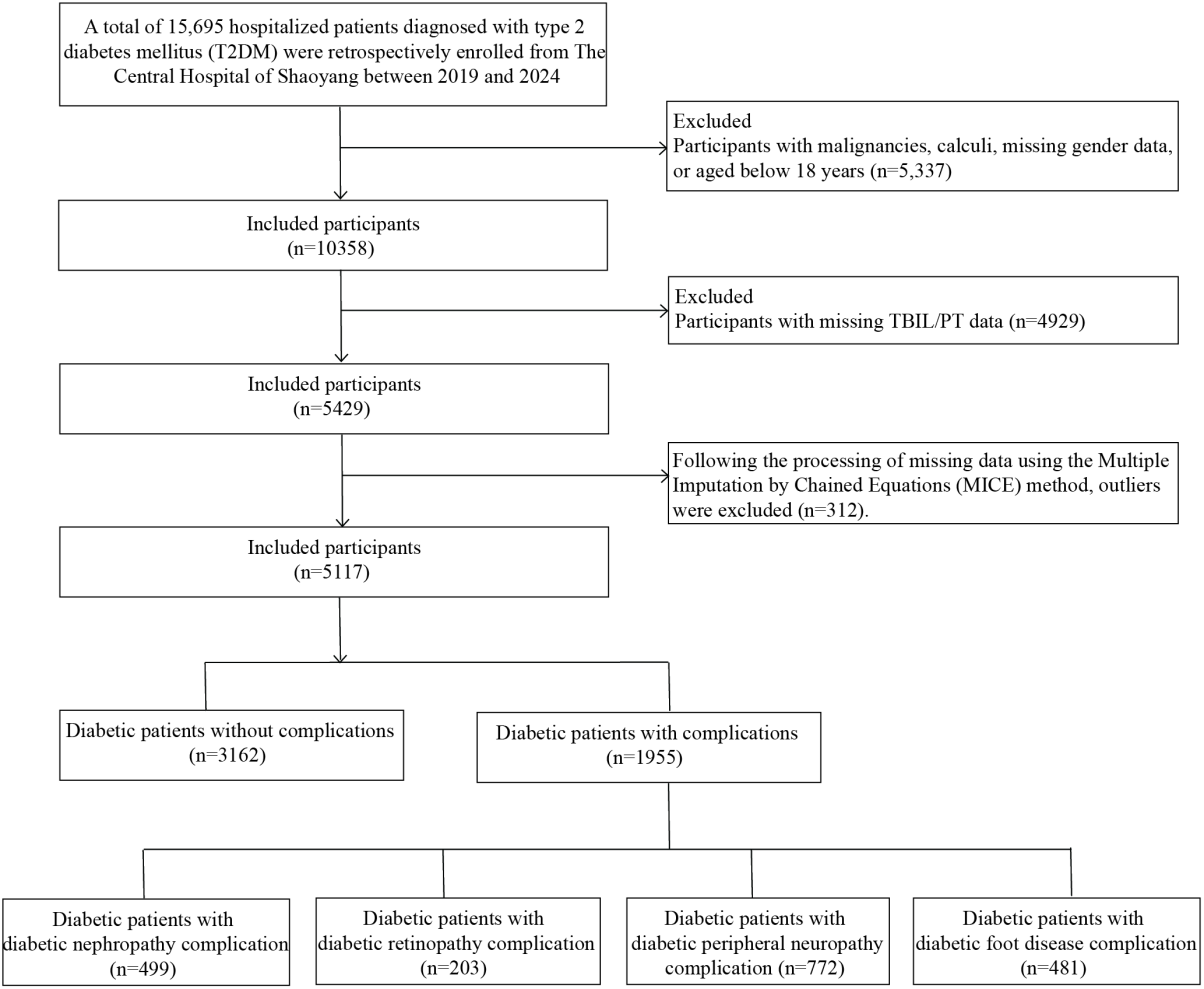
Flow chart of the selection of study participants.

This retrospective study screened 15,695 hospitalized patients with T2DM at the Central Hospital of Shaoyang between January 2019 and December 2024. The inclusion criteria were: (1) age ≥18 years; (2) confirmed T2DM duration ≥1 year; and (3) availability of complete clinical and laboratory records. Exclusion criteria comprised: (1) type 1 diabetes mellitus (T1DM) or secondary diabetes; (2) concomitant life-threatening systemic diseases, including malignancies, end-stage renal disease, severe hepatic insufficiency, or autoimmune diseases; and (3) incomplete baseline clinical data; (4) Patients with a combination of 2 or more complications. This approach was adopted to clearly analyze the independent association between TBPTRI and each complication, thereby reducing the potential confounding effects of pathophysiological interactions among different complications and their complex clinical characteristics on the association estimates. Based on these criteria, 5,337 patients were excluded, and an additional 4,929 were excluded due to missing TBIL or PT data. After applying multiple imputation by chained equations (MICE) to handle other missing values in the remaining dataset, 312 statistical outliers were further excluded, resulting in a final cohort of 5,117 eligible patients for analysis. Among them, 3,162 had no diabetic complications, while 1,955 exhibited one of four complications: diabetic nephropathy (499 cases), diabetic retinopathy (203 cases), diabetic peripheral neuropathy (772 cases), and diabetic foot ulcers (481 cases). The patient selection flowchart is presented in **Figure 1**.

### 2.2 Exposure factors

The primary exposure factor was the ratio of total bilirubin to prothrombin time, denoted as TBPTRI. TBIL (μmol/L) and PT (seconds) were measured from fasting blood samples collected within 24 hours of admission. All biochemical and coagulation analyses were performed by the hospital laboratory using standardized protocols with strict quality control procedures. TBPTRI was calculated by dividing each patient’s TBIL value by their corresponding PT value.

### 2.3 Outcome variables

The primary outcome variables of this study were the occurrence of four common chronic complications in patients with T2DM, including DN, DR, DPN, and DF. All complications were diagnosed by clinicians with intermediate or higher professional titles based on authoritative international guidelines or expert consensus and subsequently recorded in the electronic medical record system. The diagnosis of DN was established by the KDIGO 2024 Clinical Practice Guideline for Chronic Kidney Disease and the ADA-KDIGO 2022 Joint Consensus (25, 26). After excluding other kidney diseases, DN was confirmed if any of the following criteria persisted for ≥3 months: estimated glomerular filtration rate (eGFR) <60 mL/min/1.73 m^2^, urine albumin-to-creatinine ratio (UACR) ≥3 mg/mmol (equivalent to ≥30 mg/g), or other evidence of chronic kidney disease (CKD) such as abnormal urinary sediment or structural abnormalities on imaging. DR was diagnosed based on the Diabetic Retinopathy Preferred Practice Pattern (DR PPP) issued by the American Academy of Ophthalmology (AAO) (27, 28). A comprehensive evaluation was performed by ophthalmologists using dilated fundus examination, color fundus photography, fluorescein fundus angiography (FFA), or optical coherence tomography (OCT) of the retina. DPN was diagnosed according to the 2025 ADA Standards of Medical Care in Diabetes (29), incorporating clinical history, typical neuropathic symptoms (e.g., numbness, tingling, burning sensation, or hypoesthesia), and abnormal neurological signs, including 10-g monofilament testing, 128-Hz tuning fork vibration perception, Achilles tendon reflex assessment, and evaluation of temperature, pain, or pressure sensation. DF was defined per the International Working Group on the Diabetic Foot (IWGDF) criteria (30), encompassing one or more pathological conditions secondary to peripheral neuropathy and/or peripheral artery disease, such as loss of protective sensation, foot ulceration, infection, neuro-osteoarthropathy (e.g., Charcot foot), gangrene, or amputation.

### 2.4 Statistical methods

All statistical analyses in this study were performed using R software (version 4.2.2) and Python software (version 3.11.5). Continuous variables were expressed as mean ± standard deviation (Mean ± SD) or median (interquartile range) [M (P25, P75)], with their distribution types determined by the Shapiro-Wilk test. Comparisons between groups for normally distributed variables were conducted using the independent t-test or one-way analysis of variance (ANOVA), while non-normally distributed variables were analyzed using the Wilcoxon rank-sum test. Categorical variables were presented as frequency (%), and group comparisons were performed using Pearson’s Chi-squared test. Missing data were handled via MICE (m = 5), with the imputation algorithm based on the RF method (31). The imputation performance was assessed using missingness lollipop plots and density distribution plots. The final analytical dataset was derived from the average of five imputed datasets. Outliers in numerical variables were detected using the Z-score method (threshold |Z| > 5), leading to the identification and exclusion of 312 outliers. To evaluate multicollinearity among variables, variance inflation factor (VIF) calculations and Bootstrap confidence intervals (CIs) were employed, with variables exhibiting VIF > 10 considered to have significant collinearity and subsequently excluded from further analyses (32). The primary analysis employed logistic regression models to assess the association between TBPTRI and the risk of four diabetic complications. The regression models were incrementally adjusted for potential confounding factors, including age, sex, diabetes duration, Body Mass Index (BMI), fasting blood glucose, lipid levels, and liver/kidney function, with adjusted odds ratios (ORs) and their 95% CIs reported. RCS analysis was further conducted to explore potential nonlinear relationships between TBPTRI and outcome risks. Subgroup analyses and sensitivity analyses were performed to validate model robustness. In sensitivity analyses, the Synthetic Minority Over-sampling Technique (SMOTE) was applied to generate a class-balanced dataset, ensuring robustness against sample imbalance (33). This method generated synthetic minority-class samples through interpolation in feature space, implemented using Python and the imbalanced-learn library (v0.13.0), with a random seed set to 42. Additionally, three feature selection methods—LASSO regression, the Boruta algorithm, and RF variable importance ranking—were employed to screen candidate variables, with the intersection of selected variables incorporated into the final predictive model. A multivariable logistic regression model was ultimately constructed, and a nomogram was generated to visualize risk scoring. Model performance was evaluated using receiver operating characteristic (ROC) curves and the area under the curve (AUC) to assess discriminative ability. All tests were two-sided, with P < 0.05 considered statistically significant.

## 3 Results

### 3.1 Data processing and analysis of baseline information

Among the 5117 patients with T2DM who were finally included in the analysis, some of the variables were missing to varying degrees, and the distributions of missing values are shown in **Supplementary Figure 1**, among which the variables with high missing rates included HDL (24.7%), LDL (24.6%) and TG (24.2%). In order to avoid the biased effect of missing data on the results, a multiple interpolation method based on the RF algorithm (m=5) was used for processing, and the consistency of the distribution of the variables before and after interpolation was verified by a density plot (**Supplementary Figure 2**), and the results showed that the interpolation effect was good. The post-interpolation dataset was further identified by the Z-score method to identify outliers (|Z| > 5), and a total of 312 cases of abnormal individuals were excluded. Correlations between variables were analyzed by mixed variable correlation matrix (**Supplementary Figure 3**), which showed that TBPTRI was weakly correlated with most of the covariates, suggesting its independence. Multiple covariance analysis was subsequently performed (**Supplementary Table 1**), and the results showed that some of the variables had VIF values greater than 5 in the four comorbidity datasets, including AGR, TC, LDL, and GLB, suggesting the presence of moderate covariance, and the above variables had been excluded from the subsequent analysis. The results of the variable normality test are shown in **Supplementary Table 2**; most continuous variables did not obey normal distribution, and the corresponding nonparametric methods were selected for subsequent statistical analysis. Based on the cleaned dataset, the baseline characteristics of the four complication datasets —diabetic nephropathy, retinopathy, peripheral neuropathy, and podiatry —were compared separately. The results showed that patients in the diabetic nephropathy group were older, had a longer disease duration, had significantly higher renal function indicators (e.g., CREA, UA, and UREA), and had significantly lower TBPTRI (see **Table 1**); In the retinopathy group, patients’ age, disease duration, and the levels of ALT, AST, and FIB were higher than those in the group without the disease (**Supplementary Table 3**); In the peripheral neuropathy dataset, patients with combined DPN were significantly higher than controls in terms of age, disease duration, UA, and FIB, and TBPTRI was also significantly lower (**Supplementary Table 4**); In contrast, in patients with diabetic foot disease, the disease duration, inflammatory markers (e.g., WBC, CRP), and abnormalities in renal function were more pronounced. TBPTRI was also significantly decreased in patients with diabetic foot disease (**Supplementary Table 5**).

**Table 1:** Analysis table for baseline information in the diabetic nephropathy dataset.

Characteristic	Diabetic nephropathy			p-value^2^
	Overall N = 3,657^1^	No N = 3,158^1^	Yes N = 499^1^	
Age	65 (56, 73)	65 (57, 73)	63 (54, 71)	<0.001
Gender				0.260
Female	1,543 (42.19%)	1,344 (42.56%)	199 (39.88%)	
Male	2,114 (57.81%)	1,814 (57.44%)	300 (60.12%)	
Smoking				0.986
No	2,808 (76.78%)	2,425 (76.79%)	383 (76.75%)	
Yes	849 (23.22%)	733 (23.21%)	116 (23.25%)	
Drinking				0.953
no	2,715 (74.24%)	2,344 (74.22%)	371 (74.35%)	
Yes	942 (25.76%)	814 (25.78%)	128 (25.65%)	
Hypertension				<0.001
no	2,333 (63.80%)	1,934 (61.24%)	399 (79.96%)	
Yes	1,324 (36.20%)	1,224 (38.76%)	100 (20.04%)	
CHD				<0.001
no	3,185 (87.09%)	2,725 (86.29%)	460 (92.18%)	
Yes	472 (12.91%)	433 (13.71%)	39 (7.82%)	
Marriage				0.831
Married	3,007 (82.23%)	2,595 (82.17%)	412 (82.57%)	
Unmarried	650 (17.77%)	563 (17.83%)	87 (17.43%)	
BMI	24.6 (21.1, 26.9)	24.6 (21.2, 26.9)	24.6 (20.6, 27.0)	0.501
ALT	20 (14, 32)	21 (14, 33)	17 (12, 26)	<0.001
ALB	38.9 (34.8, 42.3)	39.5 (35.6, 42.7)	34.8 (29.8, 38.8)	<0.001
AST	22 (17, 31)	23 (18, 32)	20 (16, 27)	<0.001
CREA	81 (64, 135)	76 (62, 106)	357 (149, 596)	<0.001
HDL	1.15 (0.98, 1.33)	1.15 (0.98, 1.33)	1.11 (0.94, 1.33)	0.039
TG	1.64 (1.16, 2.38)	1.62 (1.15, 2.37)	1.74 (1.20, 2.48)	0.077
UA	324 (257, 408)	315 (251, 395)	387 (310, 457)	<0.001
UREA	6.5 (4.9, 10.0)	6.1 (4.7, 8.5)	13.5 (8.6, 20.0)	<0.001
TT	17.30 (16.30, 18.40)	17.30 (16.30, 18.30)	17.60 (16.40, 18.60)	0.002
DD	0.58 (0.26, 1.47)	0.54 (0.25, 1.40)	0.86 (0.41, 1.77)	<0.001
FIB	2.96 (2.41, 3.64)	2.90 (2.37, 3.57)	3.37 (2.76, 4.04)	<0.001
APTT	25.4 (23.0, 28.3)	25.4 (22.8, 28.2)	25.9 (23.4, 29.2)	0.002
HB	121 (103, 134)	123 (108, 136)	99 (84, 116)	<0.001
PLT	201 (159, 248)	203 (159, 248)	195 (159, 246)	0.367
RBC	28 (4, 61)	29 (4, 62)	26 (4, 59)	<0.001
WBC	7.04 (5.71, 8.88)	7.06 (5.71, 8.94)	7.00 (5.74, 8.46)	0.203
TBPTRI	1.05 (0.74, 1.44)	1.10 (0.80, 1.50)	0.68 (0.50, 0.95)	<0.001

^1^Median (Q1, Q3), n (%); ^2^Wilcoxon rank sum test; Pearson’s Chi-squared test.

### 3.2 Association analysis between TBPTRI and four diabetic complications

To investigate the association between TBPTRI and four chronic diabetic complications, logistic regression models were constructed in four datasets: DN, DR, DPN, and DFU. The results are presented in **Table 2**. Regression analyses were performed using TBPTRI as both a continuous variable and a standardized variable, with four progressively adjusted models: Model 1 (unadjusted), Model 2 (adjusted for basic demographic characteristics, including age, sex, smoking, drinking, hypertension, CHD, and marital status, as well as comorbidities such as hypertension and myocardial infarction, and BMI), Model 3 (further adjusted for liver and kidney function and lipid-related indices), and Model 4 (a fully adjusted model incorporating 24 covariates, including inflammatory, coagulation, and hematologic parameters). The results demonstrated that TBPTRI was significantly and inversely associated with all complications across all models. In the fully adjusted model (Model 4), each unit increase in TBPTRI was associated with a significantly reduced risk of DN (OR = 0.61, 95% CI: 0.48–0.79, P < 0.001), DR (OR = 0.53, 95% CI: 0.37–0.76, P < 0.001), DPN (OR = 0.82, 95% CI: 0.70–0.95, P = 0.009), and DF (OR = 0.52, 95% CI: 0.40–0.68, P < 0.001). Consistent results were observed when analyzing the Z-standardized TBPTRI, with the risk of each complication decreasing as the ratio increased (e.g., in Model 4, each 1-standard deviation increase in the standardized TBPTRI was associated with OR = 0.67 for DN, OR = 0.60 for DR, OR = 0.85 for DPN, and OR = 0.59 for DF, all P < 0.01).

**Table 2:** Association between TBPTRI and four diabetic complications.

Variables	Model 1		Model 2		Model 3		Model 4	
	HR(95%CI)	*p*	HR(95%CI)	*p*	HR(95%CI)	*p*	HR(95%CI)	*p*
Diabetic nephropathy								
CTI (continuous)	0.14(0.10–0.18)	<0.001	0.12(0.09–0.16)	<0.001	0.54(0.42–0.69)	<0.001	0.61(0.48–0.79)	<0.001
CTI (standardized)	0.2 (0.16–0.25)	<0.001	0.18(0.15–0.23)	<0.001	0.61(0.50–0.74)	<0.001	0.67(0.55–0.83)	<0.001
Diabetic retinopathy								
CTI (continuous)	0.49(0.37–0.66)	<0.001	0.44(0.32–0.59)	<0.001	0.48(0.34–0.68)	<0.001	0.53(0.37–0.76)	<0.001
CTI (standardized)	0.56(0.44–0.72)	<0.001	0.51(0.40–0.65)	<0.001	0.55(0.42–0.73)	<0.001	0.6(0.44–0.80)	<0.001
Diabetic peripheral neuropathy								
CTI (continuous)	0.79(0.70–0.89)	<0.001	0.72(0.63–0.82)	<0.001	0.77(0.67–0.89)	<0.001	0.82(0.70–0.95)	0.009
CTI (standardized)	0.83(0.75–0.91)	<0.001	0.77(0.70–0.86)	<0.001	0.82(0.73–0.91)	<0.001	0.85(0.76–0.96)	0.009
Diabetic foot disease								
CTI (continuous)	0.25(0.19–0.32)	<0.001	0.22(0.17–0.29)	<0.001	0.35(0.27–0.46)	<0.001	0.52(0.40–0.68)	<0.001
CTI (standardized)	0.33(0.27–0.40)	<0.001	0.3(0.24–0.37)	<0.001	0.43(0.35–0.53)	<0.001	0.59(0.48–0.73)	<0.001

Model 1, no covariates were adjusted; Model 2, adjusted for age, gender, smoking, drinking, hypertension, CHD, marriage, and BMI; Model 3, adjusted for age, gender, smoking, drinking, hypertension, CHD, Marriage, BMI, ALT, ALB, AST, CREA, HDL, TG, UA, and UREA; Model 4, adjusted for age, gender, smoking, drinking, hypertension, CHD, marriage, BMI, ALT, ALB, AST, CREA, HDL, TG, UA, UREA, TT, DD, FIB, APTT, HB, PLT, RBC, and WBC.

### 3.3 Restricted cubic spline analysis

**Figure 2:**
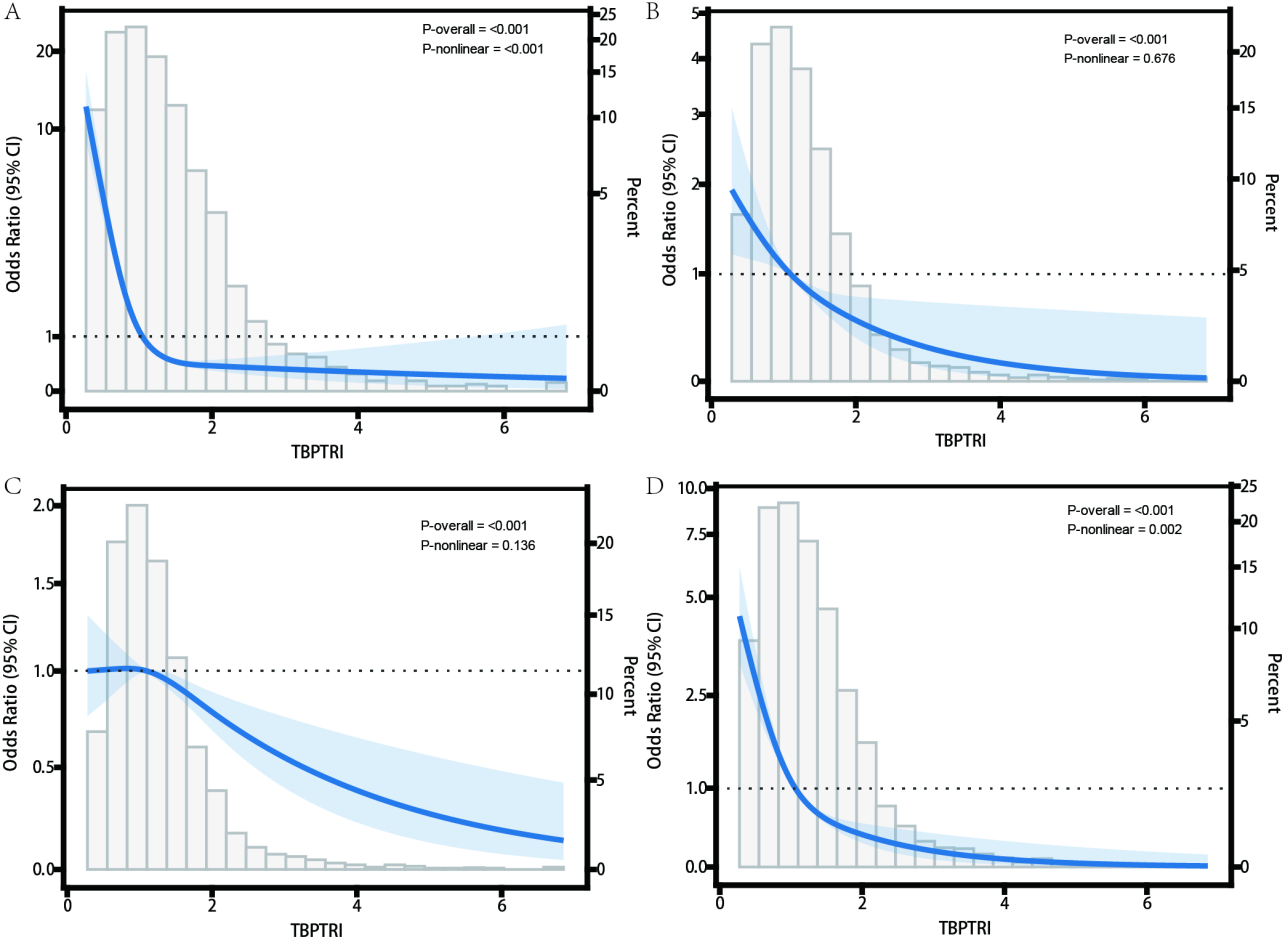
Nonlinear relationship between TBPTRI and the risk of diabetes complications. **(A)** Diabetic nephropathy dataset; **(B)** Diabetic retinopathy dataset; **(C)** Diabetic peripheral neuropathy dataset; **(D)** Diabetic foot dataset.

To further investigate the dose-response relationship between TBPTRI and diabetes-related complications, the RCS model was employed to conduct nonlinear analyses of the associations between TBPTRI and DN, DR, DPN, and DFD. The results are presented in **Figure 2**. In the diabetic nephropathy dataset (**Figure 2A**), TBPTRI exhibited a significant nonlinear inverse association with disease risk (P-overall < 0.001, P-nonlinear < 0.001). A low TBPTRI level was significantly associated with an increased risk of DN, whereas the risk declined rapidly with increasing TBPTRI and eventually plateaued. In the diabetic retinopathy dataset (**Figure 2B**), a linear inverse correlation was observed between TBPTRI and disease risk (P-overall < 0.001, P-nonlinear = 0.676), suggesting that elevated TBPTRI may reduce DR risk without a significant nonlinear trend. For diabetic peripheral neuropathy (**Figure 2C**), an inverse association was also identified (P-overall < 0.001), though the nonlinear trend was not statistically significant (P-nonlinear = 0.136). The risk reduction occurred at a relatively gradual rate, with wider confidence intervals. In the diabetic foot disease dataset (**Figure 2D**), a significant nonlinear inverse correlation was observed between TBPTRI and disease risk (P-overall < 0.001, P-nonlinear = 0.002). A low TBPTRI substantially increased the risk, followed by a rapid decline that stabilized at higher levels. The RCS analysis demonstrated that TBPTRI was inversely correlated with all examined diabetes-related complications, with particularly pronounced nonlinear patterns observed in diabetic nephropathy and diabetic foot disease.

### 3.4 Subgroup analysis

**Figure 3:**
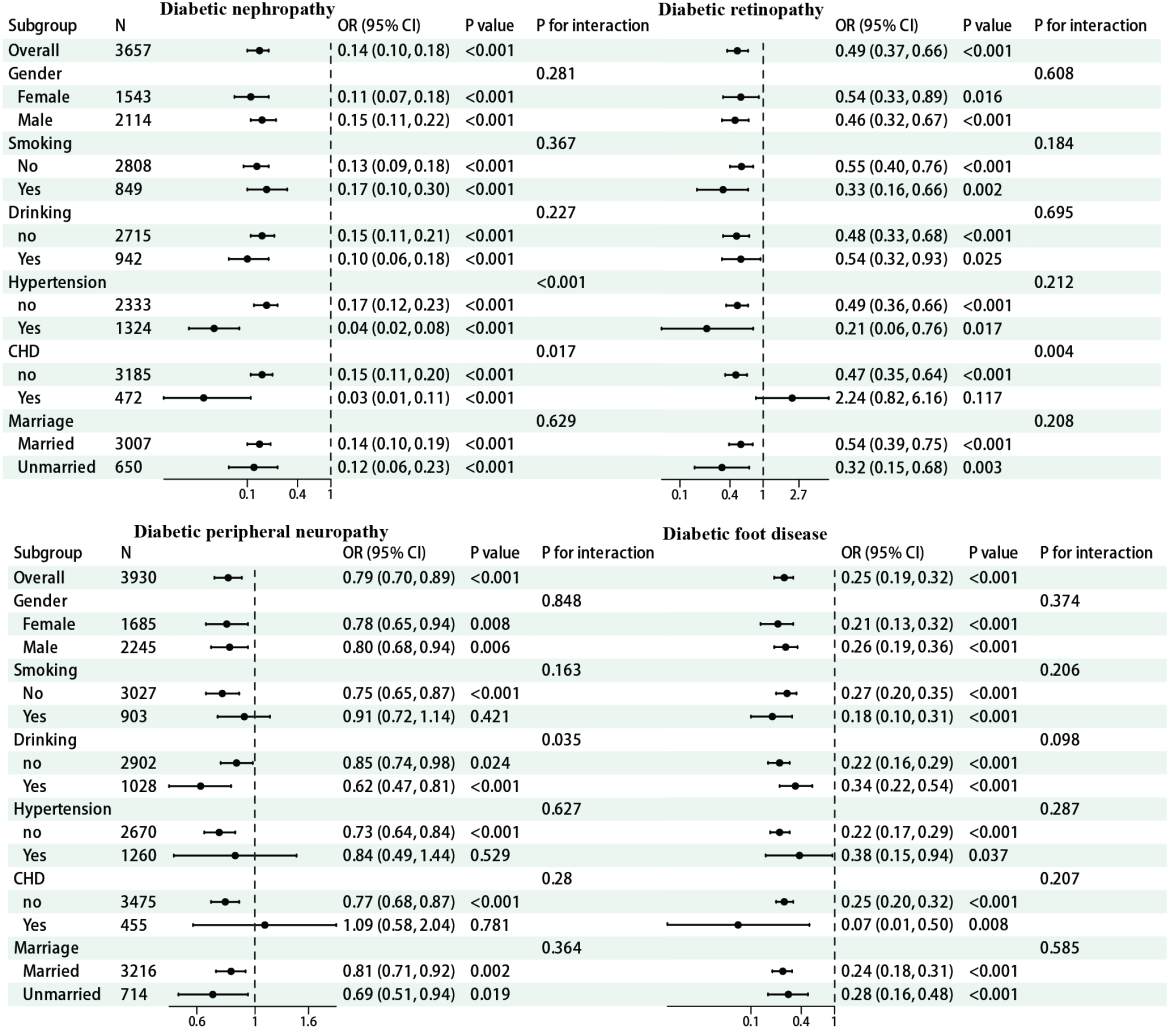
Forest map for subgroup analysis.

To evaluate the stability of the association between TBPTRI and diabetic chronic complications, as well as its heterogeneity across different populations, subgroup analyses were performed based on variables including sex, smoking status, alcohol consumption, hypertension, history of coronary heart disease (CHD), and marital status. The results are presented in **Figure 3**. In diabetic nephropathy, TBPTRI exhibited a significant protective effect across all subgroups (all P < 0.001). Notably, significant interactions were observed in individuals with or without hypertension (P for interaction < 0.001) and those with a history of CHD (P for interaction = 0.017), suggesting that hypertension and CHD history may modulate the magnitude of its effect. For diabetic retinopathy, the protective effect of TBPTRI was more pronounced in non-CHD patients (OR = 0.47, 95% CI: 0.35–0.64) and unmarried individuals (OR = 0.32, 95% CI: 0.15–0.68). A significant interaction was also detected with CHD history (P for interaction = 0.004). In diabetic peripheral neuropathy, the interaction with alcohol consumption status reached statistical significance (P for interaction = 0.035). Regarding diabetic foot, TBPTRI consistently demonstrated a negative correlation with disease risk, with no significant interactions observed in any subgroup (all P for interaction > 0.05), indicating its relatively stable effect across different populations.

### 3.5 Sensitivity analysis

To evaluate the robustness of the study results against sample imbalance, the SMOTE was employed to generate a balanced dataset (with 3,158 cases in each complication group and the control group). The association between TBPTRI and four diabetic complications was reanalyzed. Baseline characteristic analysis (**Supplementary Tables 6**–**9**) revealed that the balanced dataset retained the distribution patterns of the original data. Significant differences (all P < 0.001) were observed between the diabetic nephropathy, retinopathy, peripheral neuropathy, and foot disease groups in terms of age, sex, metabolic indicators (e.g., ALB, CREA, TG), and coagulation parameters (FIB, DD). Notably, TBPTRI was significantly lower in the complication groups than in the control group (all P < 0.001). Multivariable model analysis (**Table 3**) demonstrated that TBPTRI remained an independent protective factor across all models and for all four complications, with results highly consistent with the original analysis. In the fully adjusted model (Model 4), each unit increase in the TBPTRI was associated with a significantly reduced risk of diabetic nephropathy (OR = 0.52, 95% CI: 0.40–0.68), retinopathy (OR = 0.54, 95% CI: 0.45–0.63), peripheral neuropathy (OR = 0.75, 95% CI: 0.67–0.84), and foot disease (OR = 0.46, 95% CI: 0.39–0.55), with all P-values < 0.001. Sensitivity analysis using standardized TBPTRI yielded consistent results, with ORs ranging between 0.60 and 0.82. These findings indicate that TBPTRI maintains a stable inverse association even under balanced sample conditions, confirming the robustness of the primary conclusions and demonstrating the study’s resistance to sample imbalance.

**Table 3:** Sensitivity analysis of the relationship between TBPTRI and four diabetic complications.

Variables	Model 1		Model 2		Model 3		Model 4	
	HR(95%CI)	*p*	HR(95%CI)	*p*	HR(95%CI)	*p*	HR(95%CI)	*p*
Diabetic nephropathy								
CTI (continuous)	0.25(0.19–0.32)	<0.001	0.22(0.17–0.29)	<0.001	0.35(0.27–0.46)	<0.001	0.52(0.40–0.68)	<0.001	
CTI (standardized)	0.33(0.27–0.40)	<0.001	0.30(0.24–0.37)	<0.001	0.43(0.35–0.53)	<0.001	0.59(0.48–0.73)	<0.001	
Diabetic retinopathy								
CTI (continuous)	0.47(0.42–0.52)	<0.001	0.41(0.36–0.46)	<0.001	0.49(0.42–0.56)	<0.001	0.54(0.45–0.63)	<0.001	
CTI (standardized)	0.60(0.56–0.65)	<0.001	0.55(0.50–0.60)	<0.001	0.62(0.56–0.68)	<0.001	0.66(0.59–0.74)	<0.001	
Diabetic peripheral neuropathy								
CTI (continuous)	0.76(0.71–0.83)	<0.001	0.68(0.61–0.74)	<0.001	0.70(0.63–0.78)	<0.001	0.75(0.67–0.84)	<0.001	
CTI (standardized)	0.83(0.79–0.88)	<0.001	0.76(0.72–0.82)	<0.001	0.78(0.73–0.84)	<0.001	0.82(0.76–0.89)	<0.001	
Diabetic foot disease								
CTI (continuous)	0.22(0.19–0.25)	<0.001	0.20(0.17–0.23)	<0.001	0.32(0.27–0.37)	<0.001	0.46(0.39–0.55)	<0.001	
CTI (standardized)	0.36(0.33–0.40)	<0.001	0.34(0.30–0.37)	<0.001	0.46(0.41–0.52)	<0.001	0.60(0.53–0.67)	<0.001	

Model 1, no covariates were adjusted; Model 2, adjusted for age, gender, smoking, drinking, hypertension, CHD, marriage, and BMI; Model 3, adjusted for age, gender, smoking, drinking, hypertension, CHD, Marriage, BMI, ALT, ALB, AST, CREA, HDL, TG, UA, and UREA; Model 4, adjusted for age, gender, smoking, drinking, hypertension, CHD, marriage, BMI, ALT, ALB, AST, CREA, HDL, TG, UA, UREA, TT, DD, FIB, APTT, HB, PLT, RBC, and WBC.

### 3.6 Variable screening

**Figure 4:**
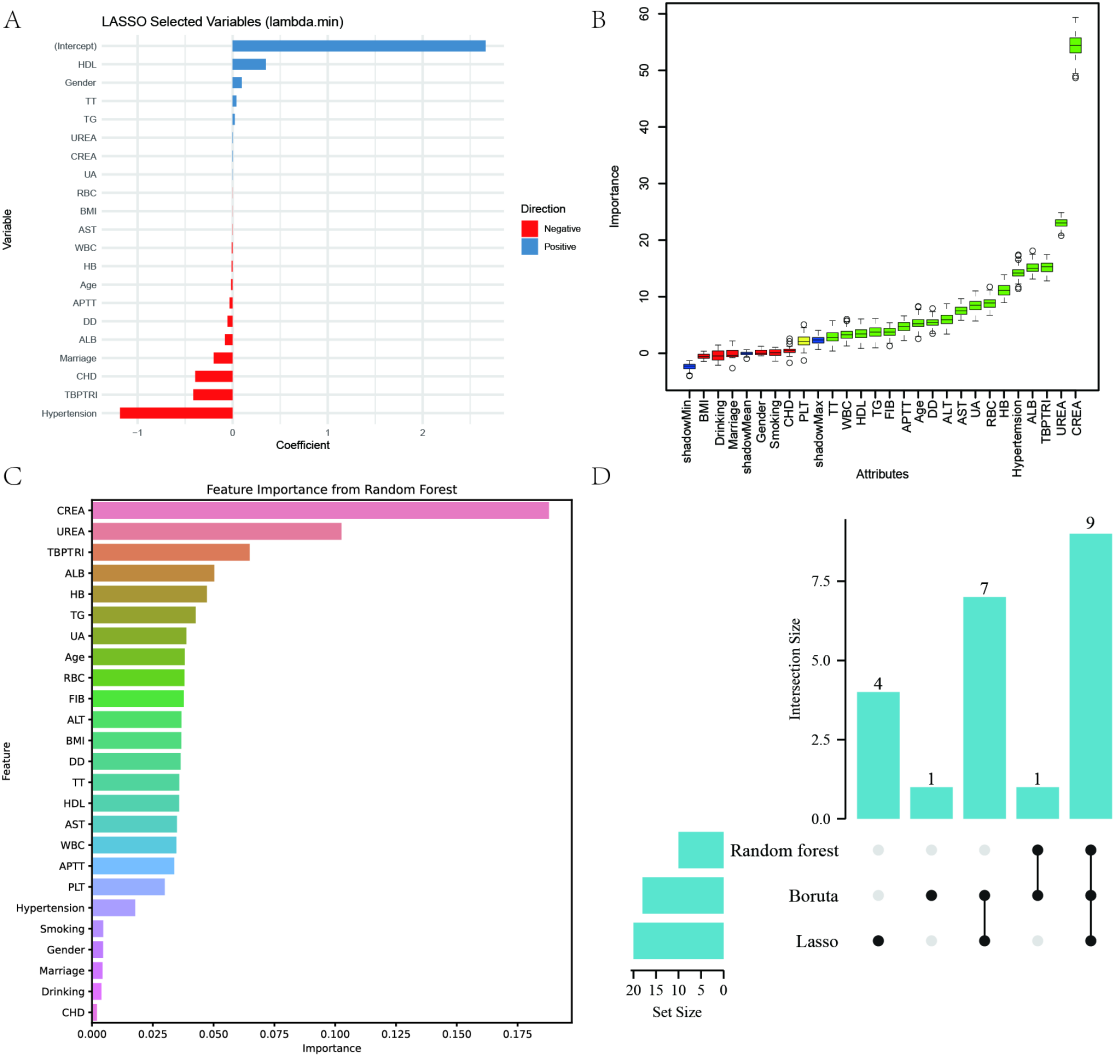
Variable selection results for the diabetic nephropathy dataset. **(A)** Key variables identified by least absolute shrinkage and selection operator (LASSO) regression; **(B)** Important variables screened using the Boruta algorithm; **(C)** Variable importance ranking derived from the Random Forest algorithm; **(D)** Venn diagram of variables selected by the three methods.

In this study, Lasso regression (lambda.min criterion), random forest (top 10 variables in terms of importance) and Boruta algorithm were used to screen the variables related to diabetic complications comprehensively, and ultimately identify the core set of predictor variables for each complication. The detailed results of the three variable selection methods are presented in **Supplementary Table 10**. The DN dataset ultimately identified nine key variables: age, ALB, CREA, TG, UA, UREA, RBC, HB, and TBPTRI (**Figure 4**). DR dataset identified 10 predictor variables such as AST, CREA, HDL, TG, UREA, DD, FIB, WBC, HB, and TBPTRI (**Supplementary Figure 4**); DPN dataset screened for Hypertension, AST, ALB, CREA, DD, RBC, PLT and TBPTRI as eight important variables (**Supplementary Figure 5**); DF dataset finalized 10 characteristic variables such as Hypertension, ALB, HDL, FIB, APTT, WBC, RBC, HB, PLT and TBPTRI (**Supplementary Figure 6**). Notably, the stable enrollment of TBPTRI as the only indicator retained in all four complication variable sets validated its core predictive value. Different complications showed specific variable combination patterns: DN was dominated by renal function indicators (CREA, UA, UREA), DR highlighted coagulation abnormalities (DD, FIB), DPN was associated with microcirculatory disorders (DD), and DF was closely associated with blood hypercoagulability (FIB, APTT).

### 3.7 Construction of ROC curves and nomograms

**Figure 5:**
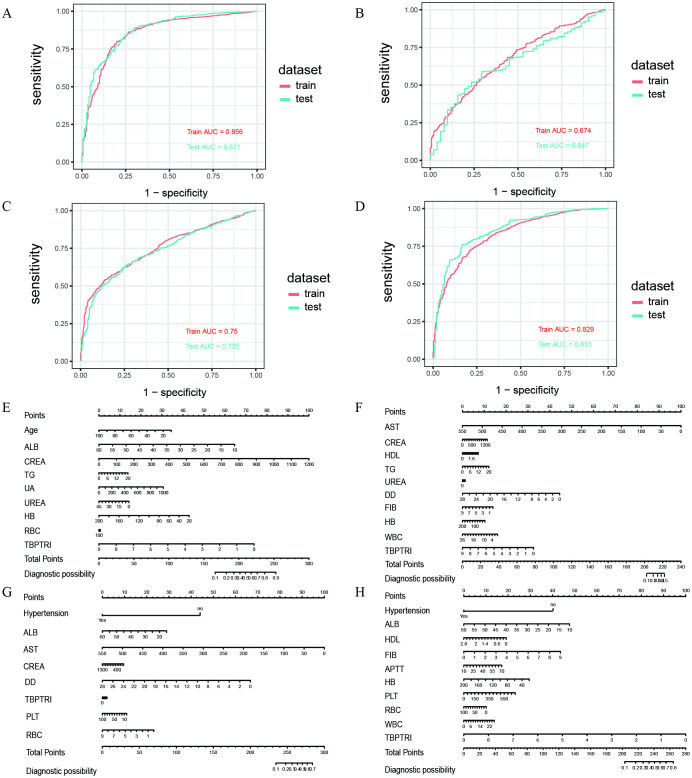
Results of ROC curve and nomogram construction. **(A–D)** ROC curves of the diabetic nephropathy (DN), diabetic retinopathy (DR), diabetic peripheral neuropathy (DPN), and diabetic foot (DF) datasets, respectively; **(E–H)** Nomograms of the corresponding datasets mentioned above.

To further evaluate the performance of the established multivariate logistic regression prediction model in discriminating the risk of diabetic complications, ROC curves were constructed for DN, DR, DPN, and DF based on the aforementioned screening results. The AUC values for both the development set and test set were calculated to validate the discriminative ability of the models, as illustrated in **Figure 5**. In the DN prediction model, the AUC values were 0.856 for the development set and 0.871 for the test set, demonstrating robust generalization performance (**Figure 5A**). For the DR prediction model, the AUC values were 0.674 (development set) and 0.647 (test set), indicating moderate discriminative capability, though slightly inferior to other models (**Figure 5B**). The DPN model exhibited AUC values of 0.750 (development set) and 0.735 (test set), suggesting consistent performance across datasets (**Figure 5C**). The DFU model achieved AUC values of 0.829 (development set) and 0.855 (test set), reflecting high predictive accuracy (**Figure 5D**). To enhance model interpretability and clinical applicability, nomograms were developed for the four diabetic complications based on the multivariate logistic regression results (**Figures 5E–H**). Each nomogram visualizes the selected core variables as a scoring system, enabling clinicians to calculate a total score based on individual patient indicators and subsequently estimate the personalized probability of developing diabetic complications. To quantitatively evaluate the incremental predictive value of TBPTRI, the performance of prediction models with and without TBPTRI was compared (**Supplementary Figure 7**). For diabetic kidney disease, the inclusion of TBPTRI improved the model’s AUC from 0.860 to 0.863. For diabetic foot disease, the AUC increased from 0.828 to 0.837. Slight improvements in AUC were also observed for diabetic retinopathy and peripheral neuropathy. The results indicate that TBPTRI can provide additional incremental information for the prediction of complication risks, particularly for kidney disease and foot disease.

## 4 Discussion

This study aimed to investigate the relationship between TBPTRI and four common complications of T2DM in patients. A retrospective analysis of 5,117 patients revealed that TBPTRI was significantly and inversely associated with the risk of multiple diabetic complications, suggesting that this ratio may serve as an independent protective factor against diabetic complications. These findings align with previous studies on the protective effects of bilirubin but innovatively integrate dual information on antioxidant and coagulation functions through TBPTRI, significantly enhancing predictive efficacy. Nonlinear analysis demonstrated a distinct threshold effect in the association between TBPTRI and diabetic nephropathy as well as diabetic foot disease, indicating the potential existence of a critical protective level. This discovery provides a potential target for clinical intervention. Notably, even after multivariate adjustment, TBPTRI retained its independent predictive value, suggesting that its mechanism of action may not entirely depend on traditional metabolic risk factors.

In terms of clinical application, the predictive model developed in this study exhibited excellent discriminative ability, particularly for nephropathy and foot disease (with AUC values of 0.871 and 0.855, respectively). Compared to existing risk scoring systems, the strength of this model lies in its incorporation of readily available routine laboratory indicators and the implementation of a visualized nomogram for convenient individualized risk assessment. These features make it particularly suitable for widespread adoption in primary healthcare settings, potentially improving the early detection rate of diabetic complications.

The clinical significance of the TBPTRI stems from its foundation in intervening in two core pathophysiological pathways of diabetic complications. TBIL and PT represent two critical and interrelated physiological processes—the antioxidant/anti-inflammatory system and the coagulation/vascular endothelial status, respectively (34, 35). Bilirubin, the end product of heme degradation, has been demonstrated to function as an endogenous antioxidant capable of scavenging superoxide anions, inhibiting lipid peroxidation, and activating antioxidant defenses by modulating the superoxide signaling pathway during neurotransmission (36, 37). In addition to directly scavenging free radicals, bilirubin can also upregulate the cellular antioxidant defense system via the Nrf2/HO-1 signaling pathway and exert significant anti-inflammatory effects by inhibiting key pro-inflammatory cytokines such as tumor necrosis factor-α (TNF-α) and interleukin-6 (IL-6). This dual action alleviates chronic low-grade inflammation and oxidative stress, which are fundamental contributors to the development of both diabetic microvascular and macrovascular injuries (38–40). On the other hand, PT, as an indicator of the extrinsic coagulation pathway, reflects coagulation activity, hepatic functional reserve, and systemic inflammation levels (41). Patients with T2DM often exhibit a hypercoagulable state, which promotes microvascular endothelial injury and thrombus formation, further exacerbating ischemic and hypoxic conditions (42, 43). Notably, while prolonged PT is typically regarded as a manifestation of coagulation dysfunction, in metabolic disorders, its elevation frequently serves as a marker of endothelial dysfunction and chronic inflammatory activation (44, 45). Therefore, the integration of TBIL and PT as a ratio provides a more comprehensive assessment of the imbalance in the oxidative stress-inflammation-coagulation interaction mechanism in T2DM patients. The innovation of the TBPTRI lies in integrating these two dimensions into a single index. While TBIL alone reflects antioxidant capacity and PT alone indicates coagulation status, the combination of the two, conceptualized as the TBPTRI, provides a more comprehensive assessment by representing the balance between “endogenous antioxidant and anti-inflammatory potential” and the “coagulation-inflammation status”.

TBPTRI demonstrated a consistent negative correlation with multiple diabetic complications, suggesting its potential involvement in multiorgan damage induced by chronic hyperglycemia through various mechanisms. Firstly, as an endogenous antioxidant molecule, TBIL mitigates oxidative stress in hyperglycemic conditions by suppressing NADPH oxidase activity, reducing reactive oxygen species (ROS) generation, and activating the Nrf2/HO-1 signaling pathway (46, 47). Consequently, it inhibits glomerulosclerosis and renal tubulointerstitial fibrosis in the kidneys, preserves blood-retinal barrier integrity in the retina, and alleviates axonal degeneration and impaired nerve conduction in peripheral nerves (48–50). Additionally, bilirubin exhibits anti-inflammatory properties by downregulating proinflammatory cytokines such as IL-6 and TNF-α, thereby ameliorating systemic chronic inflammation (51). On the other hand, PT reflects coagulation function and microcirculatory perfusion (52). Diabetic patients frequently exhibit a hypercoagulable state, which exacerbates microvascular endothelial dysfunction, capillary occlusion, and regional ischemia-hypoxia, particularly in foot wound healing and retinal microcirculatory impairment (53, 54). PT alterations serve not only as indirect indicators of hepatic function but may also signify the “second hit” role of the coagulation system in chronic complication pathogenesis (55). Thus, a decreased TBPTRI may indicate concurrent impairment of antioxidant capacity and hypercoagulability, collectively driving the initiation and progression of target organ damage in diabetic nephropathy, retinopathy, neuropathy, and foot disease through multiple pathways.

The innovation of this study lies in being the first to investigate the relationship between TBPTRI and four common chronic complications of T2DM in a large-scale population. Previous studies have primarily focused on the association between individual biochemical markers and diabetic complications, whereas this study fills a critical knowledge gap by comprehensively analyzing the ratio of TBIL to PT. Our results demonstrate that TBPTRI serves as an independent protective factor against chronic diabetic complications across multiple adjusted models, particularly exhibiting significant protective effects in diabetic nephropathy and diabetic foot disease. The findings of this study have important clinical implications. Our results suggest that TBPTRI may serve as a potential biomarker for assessing the risk of diabetic complications in patients with diabetes, enabling clinicians to optimize monitoring and management strategies. Furthermore, the constructed nomogram offers physicians a personalized risk assessment tool in clinical practice, aiding in the identification of high-risk patients and facilitating tailored interventions. This application not only improves patient prognosis but may also reduce the economic burden associated with diabetes-related complications, thereby informing the development and optimization of diabetes management policies.

Several limitations should be acknowledged. First, as a retrospective cross-sectional study, the design inherently limits the possibility of causal inference. A significant association between TBPTRI and complications was observed; however, the temporal sequence cannot be determined, and the possibility of reverse causality exists (e.g., advanced complications affecting liver function or systemic inflammation, thereby altering TBPTRI). The retrospective nature of data collection also carries a risk of selection bias, as the included hospitalized patients may represent a more severe spectrum of type 2 diabetes than the general diabetic population, which could affect the generalizability of our risk estimates. Second, missing clinical data from some patients were addressed using multiple imputation; however, this approach may not eliminate the impact of missing values. Additionally, since the data were derived from a single-center cohort, the generalizability of the findings may be limited. Future validation in larger, multi-center studies across diverse populations is warranted. Thirdly, while this cross-sectional study untangles the association between TBPTRI and diabetic complications, it cannot establish causality. Further prospective studies are needed to explore this relationship. Lastly, to clarify the independent association between TBPTRI and each complication, patients with multiple complications were excluded. This approach may limit the generalizability of the findings to diabetic patient populations with more complex clinical conditions and could introduce selection bias. In future analyses, including these patients and performing sensitivity analyses (e.g., treating multiple complications as a single group or re−analyzing after adjusting for their impact) would help verify the robustness of the core findings of this study in a broader population. This will be an important direction for our subsequent research.

## 5 Conclusions

This study highlights the potential of TBPTRI as a significant independent protective factor against chronic complications in T2DM, demonstrating a notable inverse correlation with complication risk. RCS analysis revealed a nonlinear relationship between TBPTRI and the risks of diabetic nephropathy and diabetic foot disease, suggesting that its dynamic changes should be closely monitored in clinical practice. Furthermore, the predictive model based on multivariate logistic regression and the constructed nomogram provide clinicians with an effective tool for assessing individualized complication risks in patients, offering novel insights for future diabetes management and intervention strategies.

## Data Availability

The original contributions presented in the study are included in the article/**Supplementary Material**. Further inquiries can be directed to the corresponding author.

## Supplementary material

Supplementary Figure 1 Lollipop plot of missing value distribution.

Supplementary Figure 2 Multiple interpolation density plot.

Supplementary Figure 3 Variable correlation analysis.

Supplementary Figure 4 Variable selection results for the diabetic retinopathy dataset. **(A)** Key variables identified by least absolute shrinkage and selection operator (LASSO) regression; **(B)** Important variables screened using the Boruta algorithm; **(C)** Variable importance ranking derived from the Random Forest algorithm; **(D)** Venn diagram of variables selected by the three methods.

Supplementary Figure 5 Variable selection results for the diabetic peripheral neuropathy dataset. **(A)** Key variables identified by least absolute shrinkage and selection operator (LASSO) regression; **(B)** Important variables screened using the Boruta algorithm; **(C)** Variable importance ranking derived from the Random Forest algorithm; **(D)** Venn diagram of variables selected by the three methods.

Supplementary Figure 6 Variable selection results for the diabetic foot dataset. **(A)** Key variables identified by least absolute shrinkage and selection operator (LASSO) regression; **(B)** Important variables screened using the Boruta algorithm; **(C)** Variable importance ranking derived from the Random Forest algorithm; **(D)** Venn diagram of variables selected by the three methods.

Supplementary Figure 7 Comparison of the incremental value of adding TBPTRI to prediction models. **(A)** Diabetic Nephropathy; **(B)** Diabetic Retinopathy; **(C)** Diabetic Peripheral Neuropathy; **(D)** Diabetic Foot.
