# Supplementary figures and images for: Association between the total bilirubin to prothrombin time ratio index and diabetic retinopathy, nephropathy, peripheral neuropathy, and foot disease: a retrospective study and risk prediction model construction
Source: Front Endocrinol (Lausanne). 2026 Jan 12;16:1682680. doi: 10.3389/fendo.2025.1682680 (PMC12832254; doi:10.3389/fendo.2025.1682680)

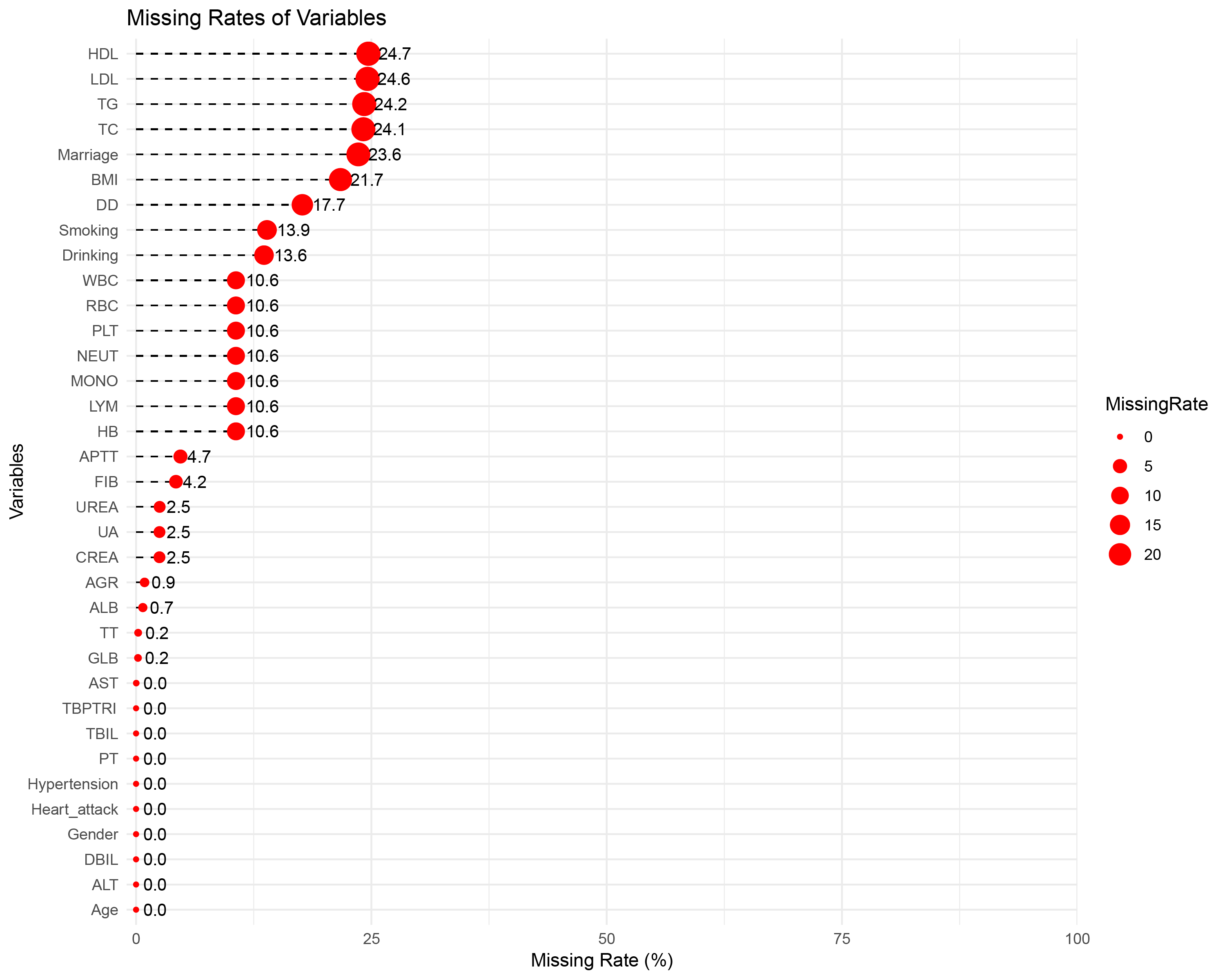

Supplement: Supplementary Figure 1 — Lollipop plot of missing value distribution. [file Image1.tif]

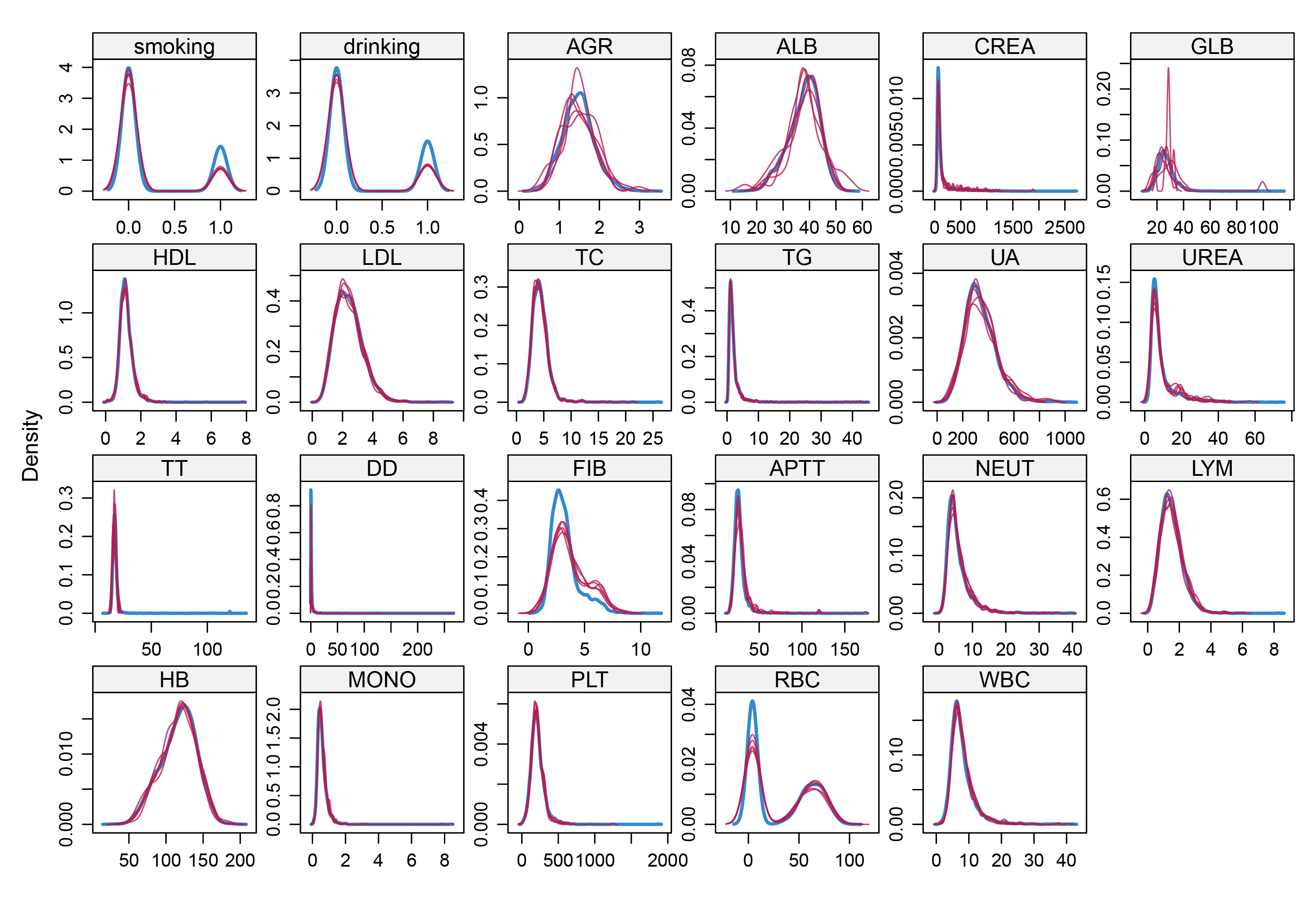

Supplement: Supplementary Figure 2 — Multiple interpolation density plot. [file Image2.tif]

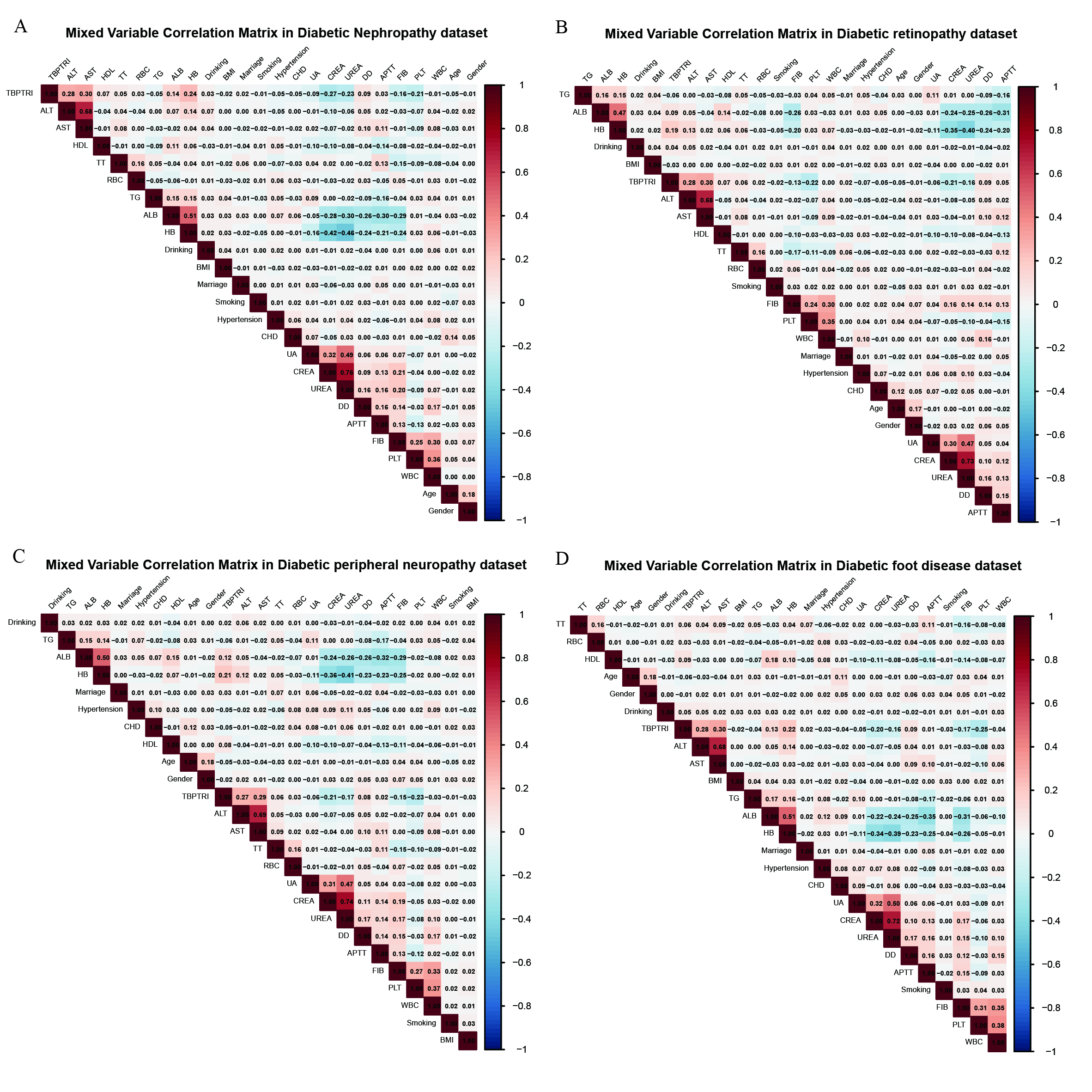

Supplement: Supplementary Figure 3 — Variable correlation analysis. [file Image3.tif]

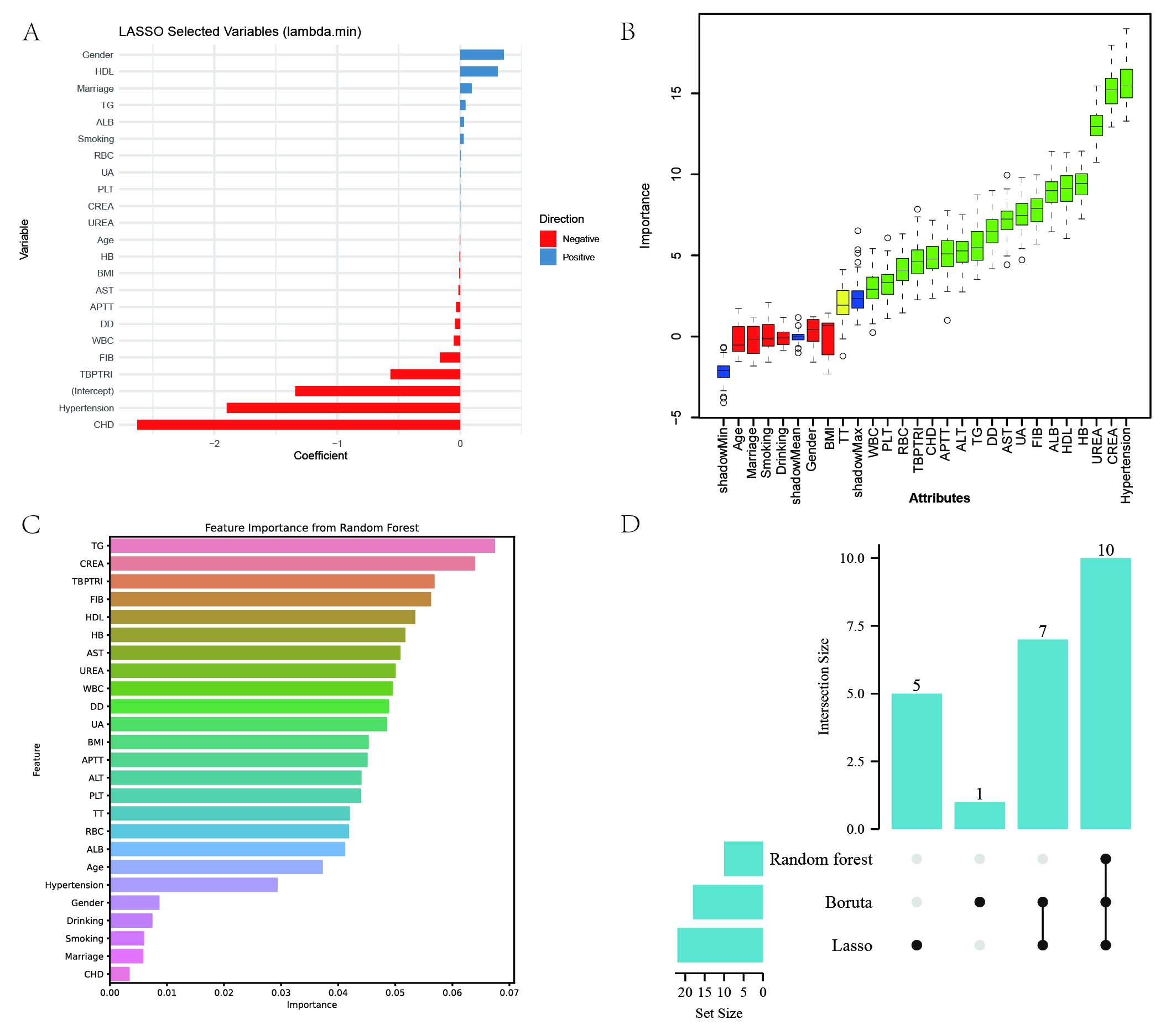

Supplement: Supplementary Figure 4 — Variable selection results for the diabetic retinopathy dataset. (A) Key variables identified by least absolute shrinkage and selection operator (LASSO) regression; (B) Important variables screened using the Boruta algorithm; (C) Variable importance ranking derived from the Random Forest algorithm; (D) Venn diagram of variables selected by the three methods. [file Image4.tif]

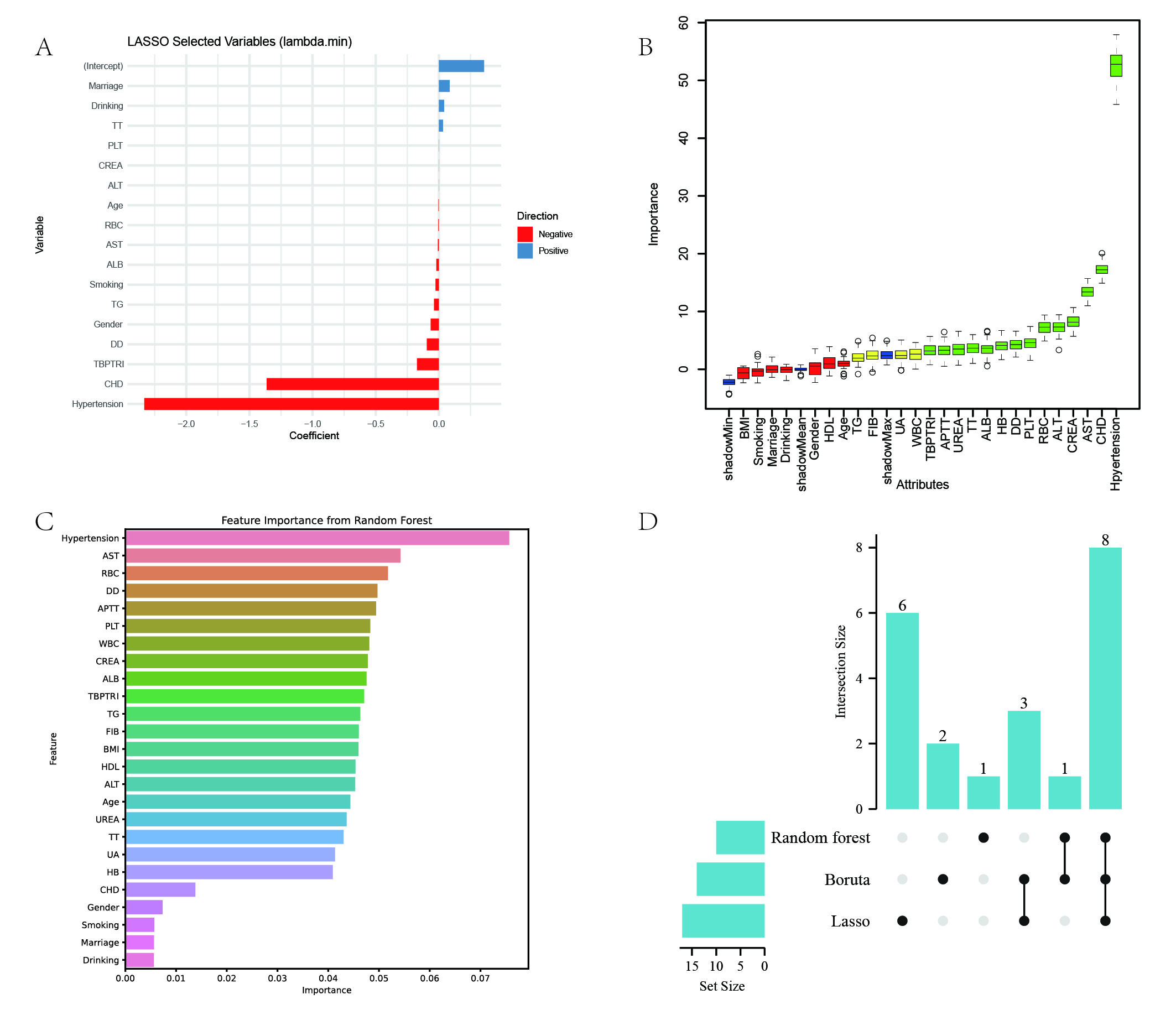

Supplement: Supplementary Figure 5 — Variable selection results for the diabetic peripheral neuropathy dataset. (A) Key variables identified by least absolute shrinkage and selection operator (LASSO) regression; (B) Important variables screened using the Boruta algorithm; (C) Variable importance ranking derived from the Random Forest algorithm; (D) Venn diagram of variables selected by the three methods. [file Image5.tif]

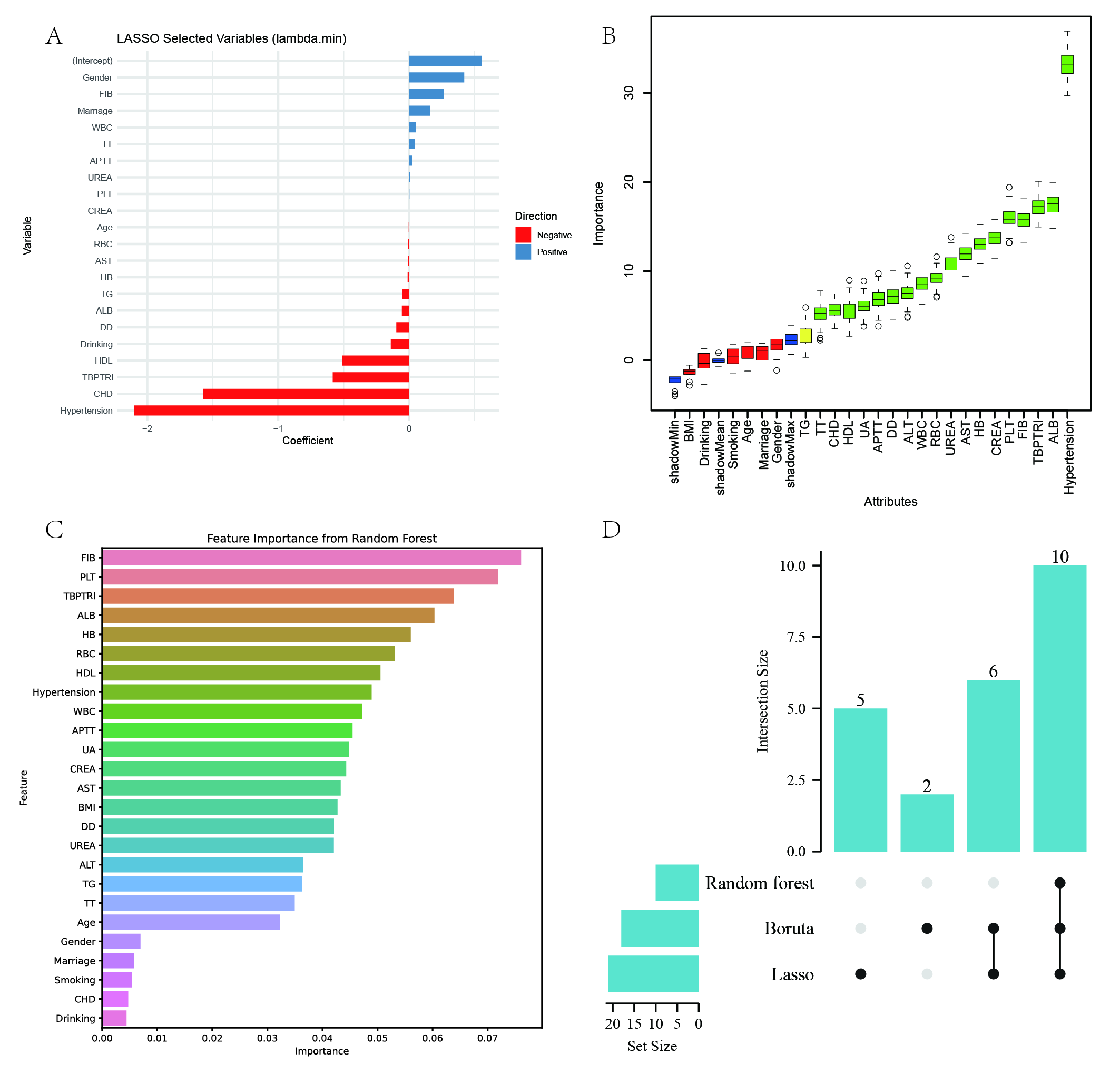

Supplement: Supplementary Figure 6 — Variable selection results for the diabetic foot dataset. (A) Key variables identified by least absolute shrinkage and selection operator (LASSO) regression; (B) Important variables screened using the Boruta algorithm; (C) Variable importance ranking derived from the Random Forest algorithm; (D) Venn diagram of variables selected by the three methods. [file Image6.tif]

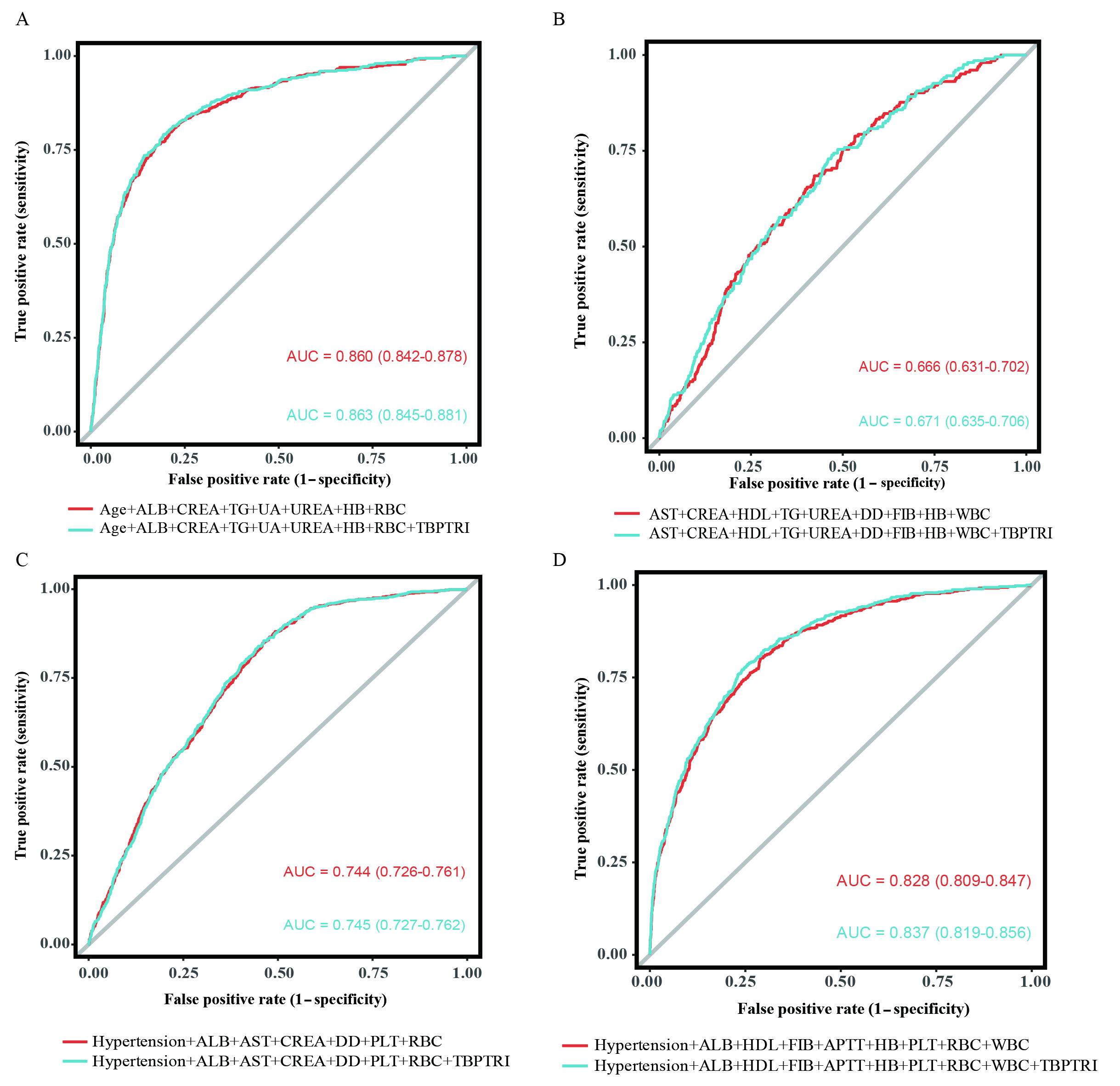

Supplement: Supplementary Figure 7 — Comparison of the incremental value of adding TBPTRI to prediction models. (A) Diabetic Nephropathy; (B) Diabetic Retinopathy; (C) Diabetic Peripheral Neuropathy; (D) Diabetic Foot. [file Image7.tif]
