# Supplementary material for: Association between the total bilirubin to prothrombin time ratio index and diabetic retinopathy, nephropathy, peripheral neuropathy, and foot disease: a retrospective study and risk prediction model construction
Source: Front Endocrinol (Lausanne). 2026 Jan 12;16:1682680. doi: 10.3389/fendo.2025.1682680 (PMC12832254; doi:10.3389/fendo.2025.1682680)
Supplement: Supplementary file 9 [file Table2.docx]

Supplementary table 2. Results of normality tests.

| **Variable** | **Test** | **Statistic** | **P_Value** |
| --- | --- | --- | --- |
| **Diabetic nephropathy dataset** | |  |  |
| Age | Lilliefors (Kolmogorov-Smirnov) | 0.055361 | 4.07E-29 |
| BMI | Lilliefors (Kolmogorov-Smirnov) | 0.098838 | 3.49E-97 |
| ALT | Lilliefors (Kolmogorov-Smirnov) | 0.243999 | <0.001 |
| ALB | Lilliefors (Kolmogorov-Smirnov) | 0.05484 | 1.52E-28 |
| AST | Lilliefors (Kolmogorov-Smirnov) | 0.272644 | <0.001 |
| CREA | Lilliefors (Kolmogorov-Smirnov) | 0.298142 | <0.001 |
| HDL | Lilliefors (Kolmogorov-Smirnov) | 0.070344 | 5.02E-48 |
| TG | Lilliefors (Kolmogorov-Smirnov) | 0.191717 | <0.001 |
| UA | Lilliefors (Kolmogorov-Smirnov) | 0.058631 | 7.77E-33 |
| UREA | Lilliefors (Kolmogorov-Smirnov) | 0.202099 | <0.001 |
| TT | Lilliefors (Kolmogorov-Smirnov) | 0.102764 | 2.60E-105 |
| DD | Lilliefors (Kolmogorov-Smirnov) | 0.284894 | <0.001 |
| FIB | Lilliefors (Kolmogorov-Smirnov) | 0.099166 | 7.53E-98 |
| APTT | Lilliefors (Kolmogorov-Smirnov) | 0.098494 | 1.73E-96 |
| HB | Lilliefors (Kolmogorov-Smirnov) | 0.055777 | 1.41E-29 |
| PLT | Lilliefors (Kolmogorov-Smirnov) | 0.064458 | 5.00E-40 |
| RBC | Lilliefors (Kolmogorov-Smirnov) | 0.275644 | <0.001 |
| WBC | Lilliefors (Kolmogorov-Smirnov) | 0.104321 | 1.25E-108 |
| TBPTRI | Lilliefors (Kolmogorov-Smirnov) | 0.142068 | 8.61E-205 |
| **Diabetic retinopathy dataset** | |  |  |
| Age | Lilliefors (Kolmogorov-Smirnov) | 0.051225 | 1.11E-22 |
| BMI | Lilliefors (Kolmogorov-Smirnov) | 0.099849 | 3.98E-91 |
| ALT | Lilliefors (Kolmogorov-Smirnov) | 0.244709 | <0.001 |
| ALB | Lilliefors (Kolmogorov-Smirnov) | 0.053463 | 8.21E-25 |
| AST | Lilliefors (Kolmogorov-Smirnov) | 0.274566 | <0.001 |
| CREA | Lilliefors (Kolmogorov-Smirnov) | 0.301702 | <0.001 |
| HDL | Lilliefors (Kolmogorov-Smirnov) | 0.071382 | 1.99E-45 |
| TG | Lilliefors (Kolmogorov-Smirnov) | 0.189803 | <0.001 |
| UA | Lilliefors (Kolmogorov-Smirnov) | 0.060514 | 3.58E-32 |
| UREA | Lilliefors (Kolmogorov-Smirnov) | 0.199039 | <0.001 |
| TT | Lilliefors (Kolmogorov-Smirnov) | 0.10202 | 2.91E-95 |
| DD | Lilliefors (Kolmogorov-Smirnov) | 0.291696 | <0.001 |
| FIB | Lilliefors (Kolmogorov-Smirnov) | 0.099048 | 1.27E-89 |
| APTT | Lilliefors (Kolmogorov-Smirnov) | 0.095828 | 1.05E-83 |
| HB | Lilliefors (Kolmogorov-Smirnov) | 0.055579 | 6.42E-27 |
| PLT | Lilliefors (Kolmogorov-Smirnov) | 0.065621 | 4.12E-38 |
| RBC | Lilliefors (Kolmogorov-Smirnov) | 0.272021 | <0.001 |
| WBC | Lilliefors (Kolmogorov-Smirnov) | 0.102759 | 1.08E-96 |
| TBPTRI | Lilliefors (Kolmogorov-Smirnov) | 0.145358 | 3.39E-197 |
| **Diabetic peripheral neuropathy dataset** | |  |  |
| Age | Lilliefors (Kolmogorov-Smirnov) | 0.049526 | 8.06E-25 |
| BMI | Lilliefors (Kolmogorov-Smirnov) | 0.100856 | 5.81E-109 |
| ALT | Lilliefors (Kolmogorov-Smirnov) | 0.246052 | <0.001 |
| ALB | Lilliefors (Kolmogorov-Smirnov) | 0.057941 | 1.74E-34 |
| AST | Lilliefors (Kolmogorov-Smirnov) | 0.274798 | <0.001 |
| CREA | Lilliefors (Kolmogorov-Smirnov) | 0.29734 | <0.001 |
| HDL | Lilliefors (Kolmogorov-Smirnov) | 0.071188 | 5.54E-53 |
| TG | Lilliefors (Kolmogorov-Smirnov) | 0.190816 | <0.001 |
| UA | Lilliefors (Kolmogorov-Smirnov) | 0.060573 | 7.66E-38 |
| UREA | Lilliefors (Kolmogorov-Smirnov) | 0.202063 | <0.001 |
| TT | Lilliefors (Kolmogorov-Smirnov) | 0.095469 | 2.65E-97 |
| DD | Lilliefors (Kolmogorov-Smirnov) | 0.294511 | <0.001 |
| FIB | Lilliefors (Kolmogorov-Smirnov) | 0.105372 | 3.01E-119 |
| APTT | Lilliefors (Kolmogorov-Smirnov) | 0.097249 | 4.40E-101 |
| HB | Lilliefors (Kolmogorov-Smirnov) | 0.05701 | 2.45E-33 |
| PLT | Lilliefors (Kolmogorov-Smirnov) | 0.07052 | 5.91E-52 |
| RBC | Lilliefors (Kolmogorov-Smirnov) | 0.289104 | <0.001 |
| WBC | Lilliefors (Kolmogorov-Smirnov) | 0.104084 | 2.87E-116 |
| TBPTRI | Lilliefors (Kolmogorov-Smirnov) | 0.140633 | 1.36E-215 |
| **Diabetic foot disease dataset** | |  |  |
| Age | Lilliefors (Kolmogorov-Smirnov) | 0.048972 | 2.15E-22 |
| BMI | Lilliefors (Kolmogorov-Smirnov) | 0.09961 | 2.89E-98 |
| ALT | Lilliefors (Kolmogorov-Smirnov) | 0.246364 | <0.001 |
| ALB | Lilliefors (Kolmogorov-Smirnov) | 0.051714 | 4.14E-25 |
| AST | Lilliefors (Kolmogorov-Smirnov) | 0.272111 | <0.001 |
| CREA | Lilliefors (Kolmogorov-Smirnov) | 0.29641 | <0.001 |
| HDL | Lilliefors (Kolmogorov-Smirnov) | 0.071347 | 3.25E-49 |
| TG | Lilliefors (Kolmogorov-Smirnov) | 0.193349 | <0.001 |
| UA | Lilliefors (Kolmogorov-Smirnov) | 0.060179 | 1.68E-34 |
| UREA | Lilliefors (Kolmogorov-Smirnov) | 0.202596 | <0.001 |
| TT | Lilliefors (Kolmogorov-Smirnov) | 0.097974 | 5.79E-95 |
| DD | Lilliefors (Kolmogorov-Smirnov) | 0.288744 | <0.001 |
| FIB | Lilliefors (Kolmogorov-Smirnov) | 0.106954 | 8.77E-114 |
| APTT | Lilliefors (Kolmogorov-Smirnov) | 0.099909 | 7.10E-99 |
| HB | Lilliefors (Kolmogorov-Smirnov) | 0.052045 | 1.90E-25 |
| PLT | Lilliefors (Kolmogorov-Smirnov) | 0.082195 | 4.87E-66 |
| RBC | Lilliefors (Kolmogorov-Smirnov) | 0.283432 | <0.001 |
| WBC | Lilliefors (Kolmogorov-Smirnov) | 0.10805 | 3.38E-116 |
| TBPTRI | Lilliefors (Kolmogorov-Smirnov) | 0.142728 | 1.01E-205 |
