# Supplementary material for: Association between the total bilirubin to prothrombin time ratio index and diabetic retinopathy, nephropathy, peripheral neuropathy, and foot disease: a retrospective study and risk prediction model construction
Source: Front Endocrinol (Lausanne). 2026 Jan 12;16:1682680. doi: 10.3389/fendo.2025.1682680 (PMC12832254; doi:10.3389/fendo.2025.1682680)
Supplement: Supplementary file 10 [file Table3.docx]

Supplementary table 3. Analysis table for baseline information in the diabetic retinopathy dataset.

| **Characteristic** | **Diabetic Retinopathy** | | | **p-value^2^** |
| --- | --- | --- | --- | --- |
|  | **Overall  N = 3,361^1^** | **No  N = 3,158^1^** | **Yes  N = 203^1^** |  |
| **Age** | 65 (57, 73) | 65 (57, 73) | 63 (56, 73) | 0.265 |
| **Gender** |  |  |  | 0.005 |
| Female | 1,410 (41.95%) | 1,344 (42.56%) | 66 (32.51%) |  |
| Male | 1,951 (58.05%) | 1,814 (57.44%) | 137 (67.49%) |  |
| **Smoking** |  |  |  | 0.643 |
| No | 2,578 (76.70%) | 2,425 (76.79%) | 153 (75.37%) |  |
| Yes | 783 (23.30%) | 733 (23.21%) | 50 (24.63%) |  |
| **Drinking** |  |  |  | 0.916 |
| no | 2,494 (74.20%) | 2,344 (74.22%) | 150 (73.89%) |  |
| Yes | 867 (25.80%) | 814 (25.78%) | 53 (26.11%) |  |
| **Hypertension** |  |  |  | <0.001 |
| no | 2,119 (63.05%) | 1,934 (61.24%) | 185 (91.13%) |  |
| Yes | 1,242 (36.95%) | 1,224 (38.76%) | 18 (8.87%) |  |
| **CHD** |  |  |  | <0.001 |
| no | 2,927 (87.09%) | 2,725 (86.29%) | 202 (99.51%) |  |
| Yes | 434 (12.91%) | 433 (13.71%) | 1 (0.49%) |  |
| **Marriage** |  |  |  | 0.304 |
| Married | 2,756 (82.00%) | 2,595 (82.17%) | 161 (79.31%) |  |
| Unmarried | 605 (18.00%) | 563 (17.83%) | 42 (20.69%) |  |
| BMI | 24.5 (21.2, 26.9) | 24.6 (21.2, 26.9) | 24.3 (21.6, 26.5) | 0.397 |
| ALT | 21 (14, 33) | 21 (14, 33) | 18 (13, 27) | <0.001 |
| ALB | 39.5 (35.7, 42.7) | 39.5 (35.6, 42.7) | 39.8 (36.4, 42.8) | 0.197 |
| AST | 22 (18, 32) | 23 (18, 32) | 21 (17, 24) | <0.001 |
| CREA | 77 (62, 109) | 76 (62, 106) | 94 (72, 151) | <0.001 |
| HDL | 1.15 (0.99, 1.33) | 1.15 (0.98, 1.33) | 1.19 (1.09, 1.30) | 0.009 |
| TG | 1.65 (1.17, 2.41) | 1.62 (1.15, 2.37) | 2.09 (1.47, 2.78) | <0.001 |
| UA | 318 (253, 398) | 315 (251, 395) | 355 (294, 421) | <0.001 |
| UREA | 6.2 (4.8, 8.7) | 6.1 (4.7, 8.5) | 7.7 (5.3, 11.0) | <0.001 |
| TT | 17.30 (16.30, 18.30) | 17.30 (16.30, 18.30) | 17.50 (16.70, 18.30) | 0.041 |
| DD | 0.54 (0.26, 1.37) | 0.54 (0.25, 1.40) | 0.60 (0.34, 1.12) | 0.403 |
| FIB | 2.90 (2.36, 3.56) | 2.90 (2.37, 3.57) | 2.89 (2.30, 3.40) | 0.192 |
| APTT | 25.4 (22.8, 28.2) | 25.4 (22.8, 28.2) | 24.9 (22.2, 27.4) | 0.017 |
| HB | 123 (107, 136) | 123 (108, 136) | 117 (103, 130) | <0.001 |
| PLT | 203 (160, 248) | 203 (159, 248) | 210 (168, 255) | 0.124 |
| RBC | 30 (4, 61) | 29 (4, 62) | 42 (4, 59) | 0.941 |
| WBC | 7.03 (5.70, 8.87) | 7.06 (5.71, 8.94) | 6.71 (5.58, 7.95) | 0.008 |
| TBPTRI | 1.09 (0.79, 1.49) | 1.10 (0.80, 1.50) | 0.92 (0.68, 1.29) | <0.001 |
| ^1^Median (Q1, Q3), n (%); ^2^Wilcoxon rank sum test; Pearson's Chi-squared test. | | | | |
