# Supplementary material for: Association between the total bilirubin to prothrombin time ratio index and diabetic retinopathy, nephropathy, peripheral neuropathy, and foot disease: a retrospective study and risk prediction model construction
Source: Front Endocrinol (Lausanne). 2026 Jan 12;16:1682680. doi: 10.3389/fendo.2025.1682680 (PMC12832254; doi:10.3389/fendo.2025.1682680)
Supplement: Supplementary file 11 [file Table4.docx]

Supplementary table 4. Analysis table for baseline information in the diabetic peripheral neuropathy dataset.

| **Characteristic** | **Diabetic Peripheral Neuropathy** | | | **p-value^2^** |
| --- | --- | --- | --- | --- |
|  | **Overall  N = 3,930^1^** | **No  N = 3,158^1^** | **Yes  N = 772^1^** |  |
| **Age** | 65 (57, 73) | 65 (57, 73) | 64 (56, 72) | 0.062 |
| **Gender** |  |  |  | 0.417 |
| Female | 1,685 (42.88%) | 1,344 (42.56%) | 341 (44.17%) |  |
| Male | 2,245 (57.12%) | 1,814 (57.44%) | 431 (55.83%) |  |
| **Smoking** |  |  |  | 0.481 |
| No | 3,027 (77.02%) | 2,425 (76.79%) | 602 (77.98%) |  |
| Yes | 903 (22.98%) | 733 (23.21%) | 170 (22.02%) |  |
| **Drinking** |  |  |  | 0.270 |
| no | 2,902 (73.84%) | 2,344 (74.22%) | 558 (72.28%) |  |
| Yes | 1,028 (26.16%) | 814 (25.78%) | 214 (27.72%) |  |
| **Hypertension** |  |  |  | <0.001 |
| no | 2,670 (67.94%) | 1,934 (61.24%) | 736 (95.34%) |  |
| Yes | 1,260 (32.06%) | 1,224 (38.76%) | 36 (4.66%) |  |
| **Heart_attack** |  |  |  | <0.001 |
| no | 3,475 (88.42%) | 2,725 (86.29%) | 750 (97.15%) |  |
| Yes | 455 (11.58%) | 433 (13.71%) | 22 (2.85%) |  |
| **Marriage** |  |  |  | 0.263 |
| Married | 3,216 (81.83%) | 2,595 (82.17%) | 621 (80.44%) |  |
| Unmarried | 714 (18.17%) | 563 (17.83%) | 151 (19.56%) |  |
| BMI | 24.5 (21.2, 26.9) | 24.6 (21.2, 26.9) | 24.4 (21.3, 26.8) | 0.803 |
| ALT | 20 (14, 32) | 21 (14, 33) | 19 (14, 27) | <0.001 |
| ALB | 39.4 (35.5, 42.5) | 39.5 (35.6, 42.7) | 38.9 (35.1, 41.9) | 0.009 |
| AST | 22 (18, 31) | 23 (18, 32) | 21 (17, 26) | <0.001 |
| CREA | 77 (62, 106) | 76 (62, 106) | 79 (62, 109) | 0.230 |
| HDL | 1.15 (0.98, 1.33) | 1.15 (0.98, 1.33) | 1.13 (0.97, 1.34) | 0.441 |
| TG | 1.60 (1.13, 2.35) | 1.62 (1.15, 2.37) | 1.51 (1.05, 2.27) | 0.002 |
| UA | 314 (250, 393) | 315 (251, 395) | 311 (248, 384) | 0.174 |
| UREA | 6.1 (4.7, 8.4) | 6.1 (4.7, 8.5) | 6.0 (4.8, 8.2) | 0.439 |
| TT | 17.40 (16.30, 18.30) | 17.30 (16.30, 18.30) | 17.50 (16.60, 18.50) | <0.001 |
| DD | 0.50 (0.24, 1.31) | 0.54 (0.25, 1.40) | 0.39 (0.20, 1.01) | <0.001 |
| FIB | 2.89 (2.35, 3.57) | 2.90 (2.37, 3.57) | 2.86 (2.32, 3.59) | 0.772 |
| APTT | 25.4 (22.9, 28.3) | 25.4 (22.8, 28.2) | 25.6 (23.2, 28.5) | 0.085 |
| HB | 123 (108, 136) | 123 (108, 136) | 122 (107, 135) | 0.307 |
| PLT | 203 (160, 248) | 203 (159, 248) | 202 (162, 253) | 0.325 |
| RBC | 18 (4, 61) | 29 (4, 62) | 5 (4, 59) | <0.001 |
| WBC | 7.00 (5.67, 8.83) | 7.06 (5.71, 8.94) | 6.70 (5.51, 8.45) | 0.003 |
| TBPTRI | 1.09 (0.79, 1.48) | 1.10 (0.80, 1.50) | 1.07 (0.77, 1.39) | 0.013 |
| ^1^Median (Q1, Q3), n (%); ^2^Wilcoxon rank sum test; Pearson's Chi-squared test | | | | |
|  | | | | |
