# Supplementary material for: Association between the total bilirubin to prothrombin time ratio index and diabetic retinopathy, nephropathy, peripheral neuropathy, and foot disease: a retrospective study and risk prediction model construction
Source: Front Endocrinol (Lausanne). 2026 Jan 12;16:1682680. doi: 10.3389/fendo.2025.1682680 (PMC12832254; doi:10.3389/fendo.2025.1682680)
Supplement: Supplementary file 12 [file Table5.docx]

Supplementary table 5. Analysis table for baseline information in the diabetic foot disease dataset.

| **Characteristic** | **Diabetic foot disease** | | | **p-value^2^** |
| --- | --- | --- | --- | --- |
|  | **Overall  N = 3,639^1^** | **No  N = 3,158^1^** | **Yes  N = 481^1^** |  |
| **Age** | 65 (57, 73) | 65 (57, 73) | 64 (56, 73) | 0.319 |
| **Gender** |  |  |  | <0.001 |
| Female | 1,504 (41.33%) | 1,344 (42.56%) | 160 (33.26%) |  |
| Male | 2,135 (58.67%) | 1,814 (57.44%) | 321 (66.74%) |  |
| **Smoking** |  |  |  | 0.662 |
| No | 2,790 (76.67%) | 2,425 (76.79%) | 365 (75.88%) |  |
| Yes | 849 (23.33%) | 733 (23.21%) | 116 (24.12%) |  |
| **Drinking** |  |  |  | 0.205 |
| no | 2,714 (74.58%) | 2,344 (74.22%) | 370 (76.92%) |  |
| Yes | 925 (25.42%) | 814 (25.78%) | 111 (23.08%) |  |
| **Hypertension** |  |  |  | <0.001 |
| no | 2,388 (65.62%) | 1,934 (61.24%) | 454 (94.39%) |  |
| Yes | 1,251 (34.38%) | 1,224 (38.76%) | 27 (5.61%) |  |
| **CHD** |  |  |  | <0.001 |
| no | 3,195 (87.80%) | 2,725 (86.29%) | 470 (97.71%) |  |
| Yes | 444 (12.20%) | 433 (13.71%) | 11 (2.29%) |  |
| **Marriage** |  |  |  | 0.423 |
| Married | 2,983 (81.97%) | 2,595 (82.17%) | 388 (80.67%) |  |
| Unmarried | 656 (18.03%) | 563 (17.83%) | 93 (19.33%) |  |
| BMI | 24.6 (21.2, 26.9) | 24.6 (21.2, 26.9) | 24.5 (21.1, 26.9) | 0.915 |
| ALT | 20 (14, 32) | 21 (14, 33) | 17 (11, 28) | <0.001 |
| ALB | 38.9 (34.9, 42.3) | 39.5 (35.6, 42.7) | 34.9 (31.0, 38.7) | <0.001 |
| AST | 22 (17, 31) | 23 (18, 32) | 20 (15, 26) | <0.001 |
| CREA | 78 (63, 110) | 76 (62, 106) | 89 (69, 129) | <0.001 |
| HDL | 1.14 (0.97, 1.31) | 1.15 (0.98, 1.33) | 1.04 (0.87, 1.19) | <0.001 |
| TG | 1.60 (1.14, 2.33) | 1.62 (1.15, 2.37) | 1.52 (1.12, 2.08) | 0.002 |
| UA | 315 (250, 397) | 315 (251, 395) | 314 (246, 404) | 0.428 |
| UREA | 6.2 (4.8, 8.7) | 6.1 (4.7, 8.5) | 6.9 (5.0, 10.1) | <0.001 |
| TT | 17.30 (16.30, 18.30) | 17.30 (16.30, 18.30) | 17.20 (16.40, 18.40) | 0.490 |
| DD | 0.57 (0.26, 1.41) | 0.54 (0.25, 1.40) | 0.78 (0.42, 1.54) | <0.001 |
| FIB | 2.98 (2.42, 3.74) | 2.90 (2.37, 3.57) | 3.73 (3.01, 4.92) | <0.001 |
| APTT | 25.6 (23.0, 28.6) | 25.4 (22.8, 28.2) | 27.3 (24.7, 30.9) | <0.001 |
| HB | 121 (105, 135) | 123 (108, 136) | 107 (91, 122) | <0.001 |
| PLT | 206 (163, 255) | 203 (159, 248) | 238 (187, 319) | <0.001 |
| RBC | 25 (4, 61) | 29 (4, 62) | 5 (4, 57) | <0.001 |
| WBC | 7.14 (5.78, 9.12) | 7.06 (5.71, 8.94) | 8.01 (6.37, 10.76) | <0.001 |
| TBPTRI | 1.06 (0.76, 1.45) | 1.10 (0.80, 1.50) | 0.79 (0.60, 1.09) | <0.001 |
| ^1^Median (Q1, Q3), n (%); ^2^Wilcoxon rank sum test; Pearson's Chi-squared test | | | | |
