# Supplementary material for: Association between the total bilirubin to prothrombin time ratio index and diabetic retinopathy, nephropathy, peripheral neuropathy, and foot disease: a retrospective study and risk prediction model construction
Source: Front Endocrinol (Lausanne). 2026 Jan 12;16:1682680. doi: 10.3389/fendo.2025.1682680 (PMC12832254; doi:10.3389/fendo.2025.1682680)
Supplement: Supplementary file 13 [file Table6.docx]

Supplementary table 6. Analysis of baseline information in the balanced diabetic nephropathy dataset.

| **Characteristic** | **Diabetic nephropathy** | | | **p-value^2^** |
| --- | --- | --- | --- | --- |
|  | **Overall  N = 6,316^1^** | **No  N = 3,158^1^** | **Yes  N = 3,158^1^** |  |
| **Age** | 64 (55, 71) | 65 (57, 73) | 62 (54, 69) | <0.001 |
| **Gender** |  |  |  | <0.001 |
| Female | 4,344 (68.78%) | 1,814 (57.44%) | 2,530 (80.11%) |  |
| Male | 1,972 (31.22%) | 1,344 (42.56%) | 628 (19.89%) |  |
| **Smoking** |  |  |  | <0.001 |
| No | 5,308 (84.04%) | 2,425 (76.79%) | 2,883 (91.29%) |  |
| Yes | 1,008 (15.96%) | 733 (23.21%) | 275 (8.71%) |  |
| **Drinking** |  |  |  | <0.001 |
| No | 5,212 (82.52%) | 2,344 (74.22%) | 2,868 (90.82%) |  |
| Yes | 1,104 (17.48%) | 814 (25.78%) | 290 (9.18%) |  |
| **Hypertension** |  |  |  | <0.001 |
| No | 4,880 (77.26%) | 1,934 (61.24%) | 2,946 (93.29%) |  |
| Yes | 1,436 (22.74%) | 1,224 (38.76%) | 212 (6.71%) |  |
| **CHD** |  |  |  | <0.001 |
| No | 5,816 (92.08%) | 2,725 (86.29%) | 3,091 (97.88%) |  |
| Yes | 500 (7.92%) | 433 (13.71%) | 67 (2.12%) |  |
| **Marriage** |  |  |  | <0.001 |
| Married | 1,445 (22.88%) | 563 (17.83%) | 882 (27.93%) |  |
| Unmarried | 4,871 (77.12%) | 2,595 (82.17%) | 2,276 (72.07%) |  |
| BMI | 24.5 (21.2, 26.8) | 24.6 (21.2, 26.9) | 24.4 (21.3, 26.7) | 0.408 |
| ALT | 19 (13, 28) | 21 (14, 33) | 17 (12, 24) | <0.001 |
| ALB | 37.0 (32.8, 40.7) | 39.5 (35.6, 42.7) | 34.8 (30.9, 38.2) | <0.001 |
| AST | 21 (17, 29) | 23 (18, 32) | 20 (16, 26) | <0.001 |
| CREA | 123 (73, 425) | 76 (62, 106) | 357 (147, 611) | <0.001 |
| HDL | 1.14 (0.98, 1.32) | 1.15 (0.98, 1.33) | 1.12 (0.97, 1.30) | 0.001 |
| TG | 1.73 (1.24, 2.47) | 1.62 (1.15, 2.37) | 1.81 (1.33, 2.57) | <0.001 |
| UA | 351 (276, 433) | 315 (251, 395) | 384 (312, 453) | <0.001 |
| UREA | 9 (6, 16) | 6 (5, 8) | 14 (9, 20) | <0.001 |
| TT | 17.40 (16.50, 18.40) | 17.30 (16.30, 18.30) | 17.55 (16.67, 18.44) | <0.001 |
| DD | 0.77 (0.34, 1.64) | 0.54 (0.25, 1.40) | 0.98 (0.49, 1.82) | <0.001 |
| FIB | 3.16 (2.58, 3.88) | 2.90 (2.37, 3.57) | 3.38 (2.85, 4.14) | <0.001 |
| APTT | 25.7 (23.4, 28.5) | 25.4 (22.8, 28.2) | 25.9 (23.8, 28.8) | <0.001 |
| HB | 111 (93, 128) | 123 (108, 136) | 100 (86, 115) | <0.001 |
| PLT | 198 (161, 246) | 203 (159, 248) | 195 (162, 244) | 0.104 |
| RBC | 28 (4, 60) | 29 (4, 62) | 27 (4, 57) | <0.001 |
| WBC | 7.07 (5.88, 8.62) | 7.06 (5.71, 8.94) | 7.08 (5.99, 8.36) | 0.714 |
| TBPTRI | 0.87 (0.61, 1.23) | 1.10 (0.80, 1.50) | 0.69 (0.52, 0.93) | <0.001 |
| ^1^Median (Q1, Q3), n (%); ^2^Wilcoxon rank sum test; Pearson's Chi-squared test. | | | | |
