# Supplementary material for: Association between the total bilirubin to prothrombin time ratio index and diabetic retinopathy, nephropathy, peripheral neuropathy, and foot disease: a retrospective study and risk prediction model construction
Source: Front Endocrinol (Lausanne). 2026 Jan 12;16:1682680. doi: 10.3389/fendo.2025.1682680 (PMC12832254; doi:10.3389/fendo.2025.1682680)
Supplement: Supplementary file 14 [file Table7.docx]

Supplementary table 7. Analysis of baseline information in the balanced diabetic retinopathy dataset.

| **Characteristic** | **Diabetic retinopathy** | | | **p-value^2^** |
| --- | --- | --- | --- | --- |
|  | **Overall N = 6,316^1^** | **No N = 3,158^1^** | **Yes N = 3,158^1^** |  |
| **Age** | 64 (57, 72) | 65 (57, 73) | 64 (57, 71) | <0.001 |
| **Gender** |  |  |  | <0.001 |
| Female | 4,542 (71.91%) | 1,814 (57.44%) | 2,728 (86.38%) |  |
| Male | 1,774 (28.09%) | 1,344 (42.56%) | 430 (13.62%) |  |
| **Smoking** |  |  |  | <0.001 |
| No | 5,355 (84.78%) | 2,425 (76.79%) | 2,930 (92.78%) |  |
| Yes | 961 (15.22%) | 733 (23.21%) | 228 (7.22%) |  |
| **Drinking** |  |  |  | <0.001 |
| No | 5,294 (83.82%) | 2,344 (74.22%) | 2,950 (93.41%) |  |
| Yes | 1,022 (16.18%) | 814 (25.78%) | 208 (6.59%) |  |
| **Hypertension** |  |  |  | <0.001 |
| No | 5,033 (79.69%) | 1,934 (61.24%) | 3,099 (98.13%) |  |
| Yes | 1,283 (20.31%) | 1,224 (38.76%) | 59 (1.87%) |  |
| **CHD** |  |  |  | <0.001 |
| No | 5,882 (93.13%) | 2,725 (86.29%) | 3,157 (99.97%) |  |
| Yes | 434 (6.87%) | 433 (13.71%) | 1 (0.03%) |  |
| **Marriage** |  |  |  | <0.001 |
| Married | 1,801 (28.51%) | 563 (17.83%) | 1,238 (39.20%) |  |
| Unmarried | 4,515 (71.49%) | 2,595 (82.17%) | 1,920 (60.80%) |  |
| BMI | 24.3 (21.5, 26.4) | 24.6 (21.2, 26.9) | 24.0 (21.8, 25.8) | <0.001 |
| ALT | 19 (14, 29) | 21 (14, 33) | 18 (14, 26) | <0.001 |
| ALB | 39.8 (36.4, 42.5) | 39.5 (35.6, 42.7) | 40.0 (37.0, 42.4) | <0.001 |
| AST | 21 (18, 27) | 23 (18, 32) | 21 (18, 24) | <0.001 |
| CREA | 83 (66, 124) | 76 (62, 106) | 92 (72, 144) | <0.001 |
| HDL | 1.18 (1.06, 1.30) | 1.15 (0.98, 1.33) | 1.20 (1.11, 1.29) | <0.001 |
| TG | 1.89 (1.35, 2.64) | 1.62 (1.15, 2.37) | 2.13 (1.61, 2.78) | <0.001 |
| UA | 336 (275, 406) | 315 (251, 395) | 354 (296, 415) | <0.001 |
| UREA | 6.8 (5.2, 9.7) | 6.1 (4.7, 8.5) | 7.7 (5.7, 10.7) | <0.001 |
| TT | 17.40 (16.54, 18.20) | 17.30 (16.30, 18.30) | 17.45 (16.76, 18.19) | <0.001 |
| DD | 0.60 (0.33, 1.20) | 0.54 (0.25, 1.40) | 0.64 (0.41, 1.12) | <0.001 |
| FIB | 2.89 (2.39, 3.44) | 2.90 (2.37, 3.57) | 2.89 (2.41, 3.33) | 0.050 |
| APTT | 25.0 (22.8, 27.4) | 25.4 (22.8, 28.2) | 24.6 (22.7, 26.8) | <0.001 |
| HB | 120 (106, 132) | 123 (108, 136) | 117 (105, 128) | <0.001 |
| PLT | 206 (166, 249) | 203 (159, 248) | 208 (174, 251) | <0.001 |
| RBC | 37 (5, 59) | 29 (4, 62) | 41 (6, 56) | <0.001 |
| WBC | 6.95 (5.82, 8.25) | 7.06 (5.71, 8.94) | 6.89 (5.90, 7.85) | <0.001 |
| TBPTRI | 1.02 (0.75, 1.37) | 1.10 (0.80, 1.50) | 0.94 (0.72, 1.27) | <0.001 |
| ^1^Median (Q1, Q3), n (%); ^2^Wilcoxon rank sum test; Pearson's Chi-squared test. | | | | |
