# Supplementary material for: Association between the total bilirubin to prothrombin time ratio index and diabetic retinopathy, nephropathy, peripheral neuropathy, and foot disease: a retrospective study and risk prediction model construction
Source: Front Endocrinol (Lausanne). 2026 Jan 12;16:1682680. doi: 10.3389/fendo.2025.1682680 (PMC12832254; doi:10.3389/fendo.2025.1682680)
Supplement: Supplementary file 15 [file Table8.docx]

Supplementary table 8. Analysis of baseline information in the balanced diabetic peripheral neuropathy dataset.

| **Characteristic** | **Diabetic peripheral neuropathy** | | | **p-value^2^** |
| --- | --- | --- | --- | --- |
|  | **Overall  N = 6,316^1^** | **No  N = 3,158^1^** | **Yes  N = 3,158^1^** |  |
| **Age** | 64 (57, 72) | 65 (57, 73) | 64 (57, 71) | <0.001 |
| **Gender** |  |  |  | <0.001 |
| Female | 4,113 (65.12%) | 1,814 (57.44%) | 2,299 (72.80%) |  |
| Male | 2,203 (34.88%) | 1,344 (42.56%) | 859 (27.20%) |  |
| **Smoking** |  |  |  | <0.001 |
| No | 5,295 (83.83%) | 2,425 (76.79%) | 2,870 (90.88%) |  |
| Yes | 1,021 (16.17%) | 733 (23.21%) | 288 (9.12%) |  |
| **Drinking** |  |  |  | <0.001 |
| No | 5,108 (80.87%) | 2,344 (74.22%) | 2,764 (87.52%) |  |
| Yes | 1,208 (19.13%) | 814 (25.78%) | 394 (12.48%) |  |
| **Hypertension** |  |  |  | <0.001 |
| No | 5,047 (79.91%) | 1,934 (61.24%) | 3,113 (98.58%) |  |
| Yes | 1,269 (20.09%) | 1,224 (38.76%) | 45 (1.42%) |  |
| **CHD** |  |  |  | <0.001 |
| No | 5,858 (92.75%) | 2,725 (86.29%) | 3,133 (99.21%) |  |
| Yes | 458 (7.25%) | 433 (13.71%) | 25 (0.79%) |  |
| **Marriage** |  |  |  | <0.001 |
| Married | 1,540 (24.38%) | 563 (17.83%) | 977 (30.94%) |  |
| Unmarried | 4,776 (75.62%) | 2,595 (82.17%) | 2,181 (69.06%) |  |
| BMI | 24.4 (21.4, 26.7) | 24.6 (21.2, 26.9) | 24.3 (21.7, 26.5) | 0.298 |
| ALT | 20 (15, 29) | 21 (14, 33) | 19 (15, 26) | <0.001 |
| ALB | 39.2 (35.6, 42.1) | 39.5 (35.6, 42.7) | 39.0 (35.5, 41.7) | <0.001 |
| AST | 21 (18, 28) | 23 (18, 32) | 21 (18, 25) | <0.001 |
| CREA | 77 (62, 106) | 76 (62, 106) | 78 (63, 106) | 0.061 |
| HDL | 1.15 (1.00, 1.33) | 1.15 (0.98, 1.33) | 1.16 (1.02, 1.32) | 0.064 |
| TG | 1.59 (1.14, 2.30) | 1.62 (1.15, 2.37) | 1.56 (1.13, 2.24) | 0.006 |
| UA | 312 (250, 388) | 315 (251, 395) | 310 (250, 378) | 0.024 |
| UREA | 6.0 (4.8, 8.2) | 6.1 (4.7, 8.5) | 6.0 (4.9, 8.0) | 0.377 |
| TT | 17.40 (16.50, 18.30) | 17.30 (16.30, 18.30) | 17.44 (16.67, 18.30) | <0.001 |
| DD | 0.48 (0.23, 1.17) | 0.54 (0.25, 1.40) | 0.42 (0.22, 1.01) | <0.001 |
| FIB | 2.89 (2.38, 3.55) | 2.90 (2.37, 3.57) | 2.88 (2.38, 3.52) | 0.940 |
| APTT | 25.6 (23.3, 28.3) | 25.4 (22.8, 28.2) | 25.7 (23.7, 28.3) | <0.001 |
| HB | 123 (108, 135) | 123 (108, 136) | 123 (108, 134) | 0.137 |
| PLT | 202 (161, 248) | 203 (159, 248) | 202 (163, 249) | 0.121 |
| RBC | 6 (4, 60) | 29 (4, 62) | 5 (4, 59) | <0.001 |
| WBC | 6.90 (5.72, 8.59) | 7.06 (5.71, 8.94) | 6.77 (5.74, 8.32) | <0.001 |
| TBPTRI | 1.09 (0.81, 1.44) | 1.10 (0.80, 1.50) | 1.09 (0.83, 1.39) | 0.030 |
| ^1^Median (Q1, Q3), n (%); ^2^Wilcoxon rank sum test; Pearson's Chi-squared test | | | | |
|  | | | | |
