# Supplementary material for: Association between the total bilirubin to prothrombin time ratio index and diabetic retinopathy, nephropathy, peripheral neuropathy, and foot disease: a retrospective study and risk prediction model construction
Source: Front Endocrinol (Lausanne). 2026 Jan 12;16:1682680. doi: 10.3389/fendo.2025.1682680 (PMC12832254; doi:10.3389/fendo.2025.1682680)
Supplement: Supplementary file 16 [file Table9.docx]

Supplementary table 9. Analysis of baseline information in the balanced diabetic foot disease dataset.

| **Characteristic** | **Diabetic foot disease** | | | **p-value^2^** |
| --- | --- | --- | --- | --- |
|  | **Overall N = 6,316^1^** | **No N = 3,158^1^** | **Yes N = 3,158^1^** |  |
| **Age** | 64 (57, 72) | 65 (57, 73) | 64 (57, 71) | 0.001 |
| **Gender** |  |  |  | <0.001 |
| Female | 4,530 (71.72%) | 1,814 (57.44%) | 2,716 (86.00%) |  |
| Male | 1,786 (28.28%) | 1,344 (42.56%) | 442 (14.00%) |  |
| **Smoking** |  |  |  | <0.001 |
| No | 5,330 (84.39%) | 2,425 (76.79%) | 2,905 (91.99%) |  |
| Yes | 986 (15.61%) | 733 (23.21%) | 253 (8.01%) |  |
| **Drinking** |  |  |  | <0.001 |
| No | 5,254 (83.19%) | 2,344 (74.22%) | 2,910 (92.15%) |  |
| Yes | 1,062 (16.81%) | 814 (25.78%) | 248 (7.85%) |  |
| **Hypertension** |  |  |  | <0.001 |
| No | 5,056 (80.05%) | 1,934 (61.24%) | 3,122 (98.86%) |  |
| Yes | 1,260 (19.95%) | 1,224 (38.76%) | 36 (1.14%) |  |
| **CHD** |  |  |  | <0.001 |
| No | 5,868 (92.91%) | 2,725 (86.29%) | 3,143 (99.53%) |  |
| Yes | 448 (7.09%) | 433 (13.71%) | 15 (0.47%) |  |
| **Marriage** |  |  |  | <0.001 |
| Married | 1,607 (25.44%) | 563 (17.83%) | 1,044 (33.06%) |  |
| Unmarried | 4,709 (74.56%) | 2,595 (82.17%) | 2,114 (66.94%) |  |
| BMI | 24.4 (21.4, 26.7) | 24.6 (21.2, 26.9) | 24.3 (21.5, 26.6) | 0.348 |
| ALT | 19 (13, 29) | 21 (14, 33) | 17 (12, 26) | <0.001 |
| ALB | 37.2 (33.1, 40.8) | 39.5 (35.6, 42.7) | 35.1 (31.5, 38.2) | <0.001 |
| AST | 21 (17, 28) | 23 (18, 32) | 20 (16, 25) | <0.001 |
| CREA | 82 (66, 116) | 76 (62, 106) | 88 (71, 127) | <0.001 |
| HDL | 1.09 (0.94, 1.25) | 1.15 (0.98, 1.33) | 1.05 (0.91, 1.18) | <0.001 |
| TG | 1.57 (1.17, 2.20) | 1.62 (1.15, 2.37) | 1.53 (1.19, 2.06) | <0.001 |
| UA | 315 (249, 400) | 315 (251, 395) | 316 (248, 403) | 0.382 |
| UREA | 6.4 (4.9, 9.2) | 6.1 (4.7, 8.5) | 6.8 (5.2, 9.9) | <0.001 |
| TT | 17.30 (16.38, 18.29) | 17.30 (16.30, 18.30) | 17.27 (16.43, 18.23) | 0.416 |
| DD | 0.73 (0.35, 1.46) | 0.54 (0.25, 1.40) | 0.86 (0.49, 1.51) | <0.001 |
| FIB | 3.33 (2.66, 4.22) | 2.90 (2.37, 3.57) | 3.78 (3.12, 4.73) | <0.001 |
| APTT | 26.4 (23.9, 29.5) | 25.4 (22.8, 28.2) | 27.4 (25.1, 30.6) | <0.001 |
| HB | 115 (98, 130) | 123 (108, 136) | 107 (93, 120) | <0.001 |
| PLT | 216 (172, 279) | 203 (159, 248) | 235 (186, 311) | <0.001 |
| RBC | 11 (4, 60) | 29 (4, 62) | 5 (4, 57) | <0.001 |
| WBC | 7.5 (6.1, 9.7) | 7.1 (5.7, 8.9) | 8.1 (6.5, 10.4) | <0.001 |
| TBPTRI | 0.94 (0.70, 1.27) | 1.10 (0.80, 1.50) | 0.82 (0.63, 1.06) | <0.001 |
| ^1^Median (Q1, Q3), n (%); ^2^Wilcoxon rank sum test; Pearson's Chi-squared test | | | | |
